# Impact of drying technologies on bioactive composition of fruit pomace: a critical review and future perspectives

**DOI:** 10.1016/j.fochx.2026.103929

**Published:** 2026-04-27

**Authors:** Ali Ali Redha, Aysun Yücetepe, Tuba Esatbeyoglu

**Affiliations:** a Department of Molecular Food Chemistry and Food Development, Institute of Food and One Health, Gottfried Wilhelm Leibniz University Hannover, Am Kleinen Felde 30, 30167 Hannover, Germany; b Department of Food Engineering, Faculty of Engineering, Aksaray University, 68100 Aksaray, Türkiye

**Keywords:** Anthocyanins, Antioxidants, Circular bioeconomy, Fruit pomace, Drying, Phenolics

## Abstract

Fruit pomaces are major underutilized by-products of the juice and wine industries, rich in polyphenols, including flavonoids, anthocyanins, and tannins, as well as functional polysaccharides such as dietary fiber and pectin. Their valorization as food ingredients depends critically on drying, which stabilizes the material while strongly influencing bioactive retention. This review synthesizes recent findings on the effects of conventional (hot-air) and emerging (vacuum, microwave-assisted, infrared, refractance window, and hybrid) drying technologies applied to apple, grape, blueberry, cherry, pear, citrus, kiwi, peach, apricot, and other berry pomaces. Across matrices, oxygen exposure and residence time emerged as more decisive than temperature alone in governing bioactive stability. Optimized hot-air drying at 60–70 °C can preserve phenolics and antioxidant activity, whereas extended or high-temperature treatments accelerate oxidative degradation. Low-oxygen and rapid-transfer techniques, particularly vacuum-based and hybrid systems, consistently enhanced retention of phenolics, anthocyanins, and antioxidant activity while maintaining fiber and pectin integrity. Overall, the findings highlight the need for matrix-specific, energy-aware drying strategies to support scalable and sustainable pomace valorization.

## Highlights

• Oxygen exposure and residence time dominate bioactive losses more than temperature alone.
• Low-oxygen and rapid drying best preserve phenolics, flavonoids, and anthocyanins.
• Pomace response to drying is matrix-specific and compound-dependent.
• Scalable low-oxygen frameworks enable sustainable pomace valorization.

## 1 Introduction

**Fig. 1:**
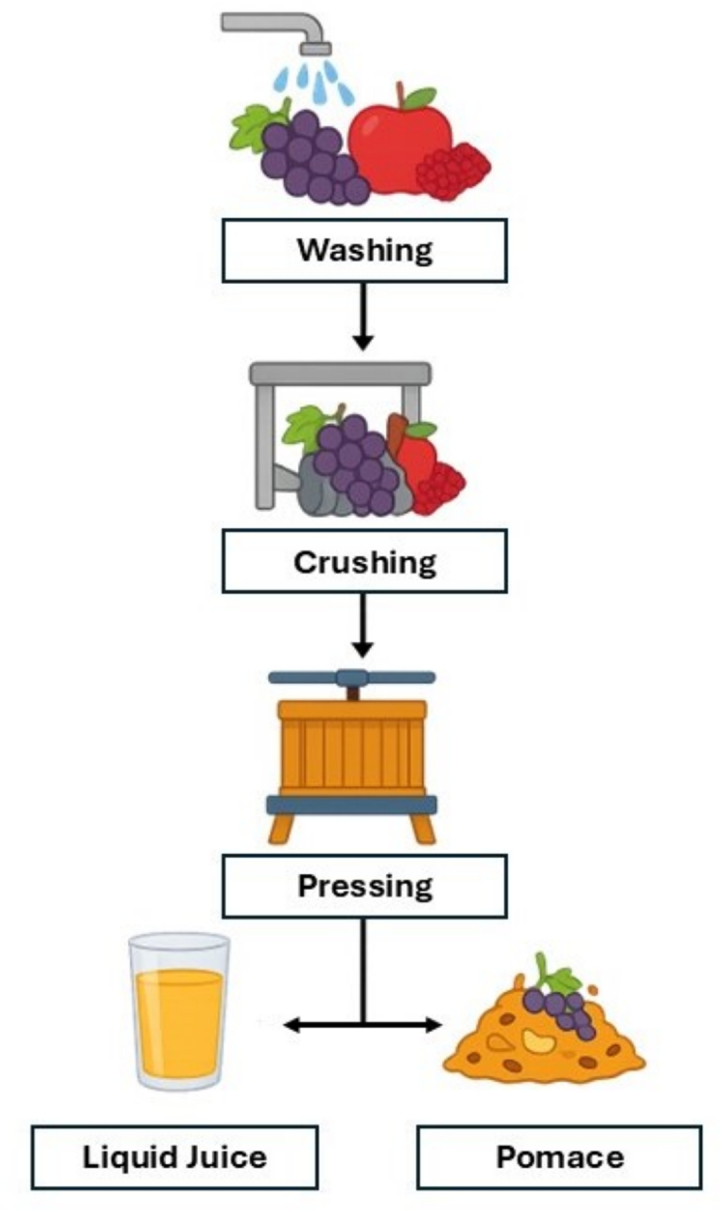
Schematic representation of the fruit juicing process, in which fresh fruits are washed, crushed, and pressed to obtain liquid juice and a solid pomace by-product (license-free icons were used to assemble the figure).

Food waste remains one of the most critical challenges facing the modern food industry, demanding coordinated action across production, processing, and consumption stages to achieve sustainable resource use. Among the major waste streams generated during food processing, fruit pomace (a by-product of juice and wine production) represents a substantial and underexploited residue. During juice extraction, fruits undergo washing, crushing, and pressing, producing two fractions: the liquid juice and a solid pomace composed mainly of peels, pulp, seeds, and stems (Fig. 1). Depending on the fruit type and processing method, pomace can represent a substantial proportion of the total fruit mass; approximately 45–60% in orange, 25% in apple, 20–30% in berries, and 20–25% in grape (Erinle & Adewole, 2022). It has been estimated that annual global production of apple pomace, for instance, is about 4 million tons (Costa et al., 2022), while grape pomace accounts for an additional 10.5–13.1 million tons worldwide (Oliveira et al., 2024).

Fruit pomace is rich in valuable bioactive and nutritional constituents, including polyphenols, flavonoids, anthocyanins, tannins, dietary fiber, and pectin, making it a promising substrate for functional ingredient recovery within a circular bioeconomy framework (Tama & Karaś, 2025). However, immediately after pressing, pomace exhibits high moisture content (typically 70–80%), which promotes microbial spoilage, enzymatic degradation, and fermentation, rendering it unsuitable for direct use or long-term storage (Kumar et al., 2024). Therefore, drying is a critical stabilization step, enabling preservation, easy handling, milling into powder, and extraction of target compounds.

From a sustainability standpoint, drying must be optimized not only for efficiency and energy consumption but also for product quality. The process should balance energy efficiency (aligning with Sustainable Development Goal 12: Responsible Consumption and Production) with nutritional and functional preservation (supporting Sustainable Development Goal 3: Good Health and Well-Being). In this context, selecting appropriate drying technologies and conditions is critical to ensure that the process remains both environmentally responsible and conducive to retaining the maximum possible levels of phenolic and other bioactive compounds. Among these, polyphenols, a chemically diverse class with documented antioxidant, anti-diabetic, anti-inflammatory, and antimicrobial activities, have received particular attention as key quality markers and functional indicators of dried fruit pomace (Raczkowska & Serek, 2024).

While general trends in phenolic retention may appear comparable across fruit pomaces, the specific phenolic composition of each matrix differs noticeably, making its response to drying highly fruit-dependent. Consequently, the influence of drying techniques and operating conditions (e.g., temperature in convective drying or power level in microwave drying) cannot be universally generalized. Fruits with high anthocyanin content, such as berries, tend to be more sensitive to heat. Anthocyanins are highly susceptible to degradation by heat, light, pH changes, and oxygen (Enaru et al., 2021). Their breakdown involves structural changes such as the opening of the flavylium ring, hydrolysis of glycosidic bonds, ring cleavage, and polymerization, all of which lead to color loss and reduced antioxidant activity. In contrast, fruit pomaces where flavonols and flavan-3-ols are the dominant polyphenols, such as those from apples and grapes, exhibit moderate heat sensitivity (Gao et al., 2022). As a result, drying conditions may not significantly affect their antioxidant properties to the same extent as in anthocyanin-rich fruits. On the other hand, phenolic acids, which are relatively less heat-sensitive and are present in many of the aforementioned fruits, tend to be more stable during thermal processing (Babaei Rad et al., 2025). In some cases, their measurable concentration may even increase due to the release of bound forms from the cell wall or esterified complexes. Therefore, the effect of drying techniques, and the specific parameters applied within those techniques, should not be assumed to have uniform impacts across all types of fruit pomace. Each pomace type must be studied in the context of its unique phenolic profile and the desired outcome for the dried product.

This literature review aims to comprehensively and critically evaluate current findings on both conventional and emerging drying technologies applied to fruit pomaces, with a particular focus on their impact on the bioactive composition. The objective is to better understand the suitability of different drying methods for various types of fruit pomace and to identify existing research gaps. In addition, this review seeks to provide recommendations for future studies on drying fruit pomace, with the goal of enhancing the quality of research outcomes that contribute to both nutritional value and sustainability.

## 2 Overview of conventional and emerging drying techniques

### 2.1 Conventional drying techniques

Drying is the most fundamental and energy-intensive step in stabilizing high-moisture food residues such as fruit pomaces, as it prevents microbial spoilage and facilitates further valorization. Among conventional methods, solar and convective drying dominate due to their technical simplicity and long-standing industrial use.

Solar drying (SR), among the earliest preservation methods, utilizes solar radiation for moisture removal either through direct exposure or via heated air from solar collectors. The process combines radiative and convective heat transfer to evaporate surface moisture (Nnamchi et al., 2025). Although inexpensive and environmentally sustainable, solar drying lacks precise process control and is highly weather-dependent, leading to long drying times and risk of contamination. Given the high moisture content and thermolabile nature of fruit pomaces, this method is generally unsuitable for their stabilization, though it may still hold value for small-scale or low-resource applications.

Convective drying, specifically as hot-air drying (HAD), remains the most widely applied industrial method for dehydrating fruit pomaces due to its reproducibility, scalability, and well-characterized kinetics. The process is governed by simultaneous heat and mass transfer phenomena, in which heated air serves as both the thermal energy carrier and the moisture removal medium (Arpaci et al., 2024). Moisture diffusion occurs in two distinct phases: an initial constant-rate period, dominated by surface water evaporation controlled by external conditions, followed by a falling-rate period, where internal capillary and diffusive transport become the rate-limiting mechanisms (Castro et al., 2018). The onset of internal resistance during this transition determines the final drying kinetics and is closely linked to the preservation of bioactive compounds and structural integrity.

Drying behavior is highly sensitive to air temperature, velocity, relative humidity, and bed thickness, as these parameters govern both the external convective flux and the internal moisture gradient (Arpaci et al., 2024; Castro et al., 2018). Increasing temperature and decreasing humidity markedly accelerate evaporation, while excessive air velocity yields diminishing returns once internal diffusion becomes limiting. Moreover, product geometry and load density significantly affect the uniformity of heat distribution and the extent of shrinkage, influencing the retention of phenolics and anthocyanins. Optimization therefore involves maintaining a sufficient temperature gradient to sustain mass flux while avoiding oxidative and polymerization reactions at elevated temperatures. Recent advances in multistage and optimized convective systems, often combining mild initial heating with intensified final phases, have improved throughput while mitigating quality losses (V. P, 2020). Although energy-intensive compared with emerging alternatives, HAD remains the benchmark for evaluating new drying technologies, supported by extensive kinetic data, established modeling frameworks, and broad industrial applicability (Hii et al., 2021; V. P, 2020).

### 2.2 Emerging drying techniques

Vacuum-based drying technologies have gained increasing attention as alternatives to conventional hot-air systems due to their ability to reduce oxidation and thermal degradation while improving mass transfer efficiency (Keçeli et al., 2026). Among these, freeze-drying (FD) or lyophilization, remains the gold standard for preserving the structural integrity and nutritional quality of fruit-derived materials. By sublimating ice directly under low pressure, FD minimizes the loss of heat-sensitive compounds and preserves color, texture, and rehydration capacity (Al Faruq et al., 2025; Nowak & Jakubczyk, 2020). However, its high operational cost, long cycle time, and energy demands have motivated the development of more sustainable vacuum-assisted and hybrid systems. Vacuum drying (VD), which removes water through evaporation at sub-atmospheric pressure, lowers the boiling point of water and thus enables drying at lower product temperatures, helping to limit oxidative and enzymatic reactions (Wardhani et al., 2021). Building on these principles, microwave-vacuum drying (MWVD) and vacuum-belt drying (VBD) integrate volumetric microwave heating or conductive contact heat with vacuum conditions to accelerate moisture migration while maintaining low thermal loads, achieving product qualities comparable to freeze-drying but with markedly reduced energy input and process time (Al Faruq et al., 2025; Wardhani et al., 2021).

Microwave drying (MD) also represents one of the most advanced and efficient thermal dehydration methods for fruit-based materials. Unlike convective systems that rely on external heat transfer, MD operates through volumetric heating, where electromagnetic waves (commonly 915 or 2450 MHz) penetrate the material and cause rapid oscillation of polar molecules, generating heat internally throughout the matrix (Calin-Sanchez et al., 2020). This inside-out heating promotes uniform temperature distribution, accelerates moisture migration, and significantly shortens drying time compared with conventional hot-air systems. The technique's volumetric energy transfer reduces both energy consumption and nutrient degradation, leading to improved retention of phenolics, vitamins, and antioxidants (Putra et al., 2025). However, MD's efficiency depends strongly on power density and product geometry; excessive energy input can lead to localized overheating or case hardening due to nonuniform field distribution. To address these drawbacks, intermittent or hybrid configurations, such as microwave–vacuum or microwave-assisted convective drying, are increasingly employed, allowing better control of mass transfer while minimizing oxidative stress on heat-sensitive compounds (Calin-Sanchez et al., 2020; Putra et al., 2025).

Infrared (IR) drying is another technique that has emerged as an efficient radiative technique offering rapid and uniform energy transfer through electromagnetic radiation, primarily within the wavelength range of 0.75–100 μm (Anumudu et al., 2024). Water molecules within the product absorb IR energy, which is then converted into heat, promoting moisture diffusion from the interior to the surface (Salehi, 2020). Compared with conventional convective drying, IR systems offer higher heat transfer rates, shorter drying times, and improved energy efficiency, often exceeding 80%, while limiting structural collapse and oxidative degradation (Zartha Sossa et al., 2021). These advantages make IR particularly suitable for thin-layer materials such as fruit pomaces, where rapid dehydration helps preserve phenolic and pigment compounds sensitive to prolonged heating. Furthermore, IR is frequently coupled with hot-air or vacuum drying to achieve synergistic enhancement in drying kinetics and bioactive retention through simultaneous convective and radiative heat transfer (Salehi, 2020).

Following the advances in radiative systems such as infrared drying, refractance window drying (RWD) has gained recognition as a fourth-generation thin-film drying technology capable of achieving rapid dehydration under mild thermal conditions while retaining high product quality (Karadbhajne et al., 2019). In this process, a thin layer of fruit puree or pomace is spread over a transparent polymer film floating on a circulating hot-water surface (typically 95–97 °C) (Waghmare, 2021). Heat transfer occurs primarily through conduction and convection, supported by limited infrared radiation from the water surface. Moisture within the product forms a transient “refractance window”, which allows thermal energy to penetrate efficiently, accelerating evaporation without substantial overheating. As the product surface dries, the window closes, self-regulating the heat flux and preserving temperature-sensitive compounds (Waghmare, 2021). Comparative studies have demonstrated that RWD yields comparable or superior retention of phenolics, carotenoids, anthocyanins, and ascorbic acid relative to conventional convective or even freeze-drying, while cutting energy use by up to 50% (Borse et al., 2025; Mahanti et al., 2021). With its low capital demand, short drying time, and strong alignment with clean-energy and waste-reduction goals, RWD offers a highly promising route for the sustainable valorization of fruit pomaces where bioactive preservation is critical.

The integration of complementary energy sources has driven the evolution of hybrid and sequential drying systems, which aim to combine the efficiency of advanced techniques with the product quality retention of milder processes (Bari et al., 2026). Hybrid drying typically merges two or more mechanisms, such as convective air with microwave, infrared, or vacuum heating, to accelerate heat and mass transfer while minimizing thermal degradation. Compared with single-stage convective systems, hybrid designs can shorten drying times by 30–70% and improve energy efficiency by up to 50%, primarily due to enhanced moisture diffusion and reduced surface hardening (Hii et al., 2021). Sequential drying approaches, in which a moderate pre-drying phase is followed by a rapid finishing step (e.g., convective followed by microwave or infrared), have been shown to improve the retention of phenolics and anthocyanins in fruit matrices. Recent analyses demonstrate that integrating forced-convection systems with microwave or vacuum modules promotes uniform heat distribution and moisture removal, yielding superior stability of heat-sensitive bioactives (Asrate & Ali, 2025). Similarly, solar-thermal hybrids equipped with auxiliary electrical or thermal energy storage units enable continuous operation under variable weather conditions, enhancing process reliability and overall product uniformity (Kong et al., 2024). Collectively, hybrid and sequential systems represent a promising route toward sustainable pomace valorization, offering a balance between process efficiency, energy conservation, and bioactive preservation.

## 3 Conventional and emerging drying techniques for fruit pomaces

A structured literature review was conducted to systematically capture current knowledge on the influence of drying technologies on fruit pomace composition. The search was performed on 21 October 2025 using Google Scholar, Scopus, Web of Science, and PubMed databases, without date restrictions to ensure comprehensive coverage of the available literature. Keyword combinations included “drying”, “dehydration”, “fruit pomace”, “press cake”, “polyphenols”, “phenolic compounds”, “flavonoids”, and “anthocyanins”. Studies were considered eligible if they examined fruit pomace or press cake and evaluated either (i) the influence of processing parameters within a given drying technique (e.g., temperature, air velocity, pressure, or power), or (ii) the comparative performance of at least two drying methods. Emphasis was placed on studies reporting total phenolic content (TPC), total flavonoid content (TFC), total anthocyanin content (TAC), and antioxidant activity as primary indicators of bioactive stability, while additional compositional attributes (e.g., dietary fiber, pectin, and individual phenolic compounds) were incorporated where relevant. Studies not involving fruit-derived pomaces or lacking quantitative assessment of bioactive composition were excluded.

### 3.1 Apple pomace

Apple (*Malus domestica*) is a globally cultivated, water-rich fruit known for its carbohydrate content (9.5–22.0%), including natural sugars and fiber (Kauser et al., 2024). The apple pomace resulting from apple processing corresponds to 25–30% of the apples' total weight (Costa et al., 2025). Although its pomace is a rich source of fiber (36.8%) and pectin (10–15%), it also contains polyphenolic compounds such as quercetin glycosides (52–68 mg/100 g dry mass, DM), procyanidin B2 (21.9 mg/100 g DM), and cinnamic acid (17.6 mg/100 g DM) (Kauser et al., 2024). Multiple studies have investigated the effects of conventional and emerging drying strategies on apple pomace properties (especially polyphenolic compounds), the findings of those studies have been summarized in Table 1.

**Table 1:** Summary of studies investigated the effect of different drying techniques on the bioactive properties of apple pomace.

Apple pomace origin	Drying technique and methodology	Key findings	Reference
Mexico	Hot air drying (cabinet) for blanched and unblanched pomace. Temperature: 50, 60, 70, and 80 °C. Relative humidity: 46%. Air velocity: 3 m/s. Final moisture: 5%. Sample weight: 1 kg.	• Following drying at 50–80 °C, both blanched and unblanched pomace showed notable decreases in TPC, TFC, and antioxidant capacity. • Anthocyanins completely degraded and became undetectable under all drying conditions. • High levels of polyphenols, flavonoids, and antioxidant activity were still present in blanched pomace that was dried at 80 °C, suggesting that blanching protected heat-sensitive compounds during drying.	Heras-Ramírez et al. (2011)
Mexico	Vacuum drying. Temperature: 32.8–73.7 °C. Sample weight: 5 g. Vacuum pressure: from 27 to 56 kPa. Drying time: from 3.32 to 6.68 h.	• An increase in drying duration caused a decrease in TPC of apple pomace. • Long drying duration and high drying temperature caused a decrease in antioxidant activity.	Almeida-Trasviña et al. (2014)
Hungary	Hot air drying. Temperature: 60 and 80 °C. Sample weight: 200 g. Drying time: 3 h at 80 °C and 6 h at 60 °C. Final moisture: ∼3–4%. Combined conventional-vacuum oven drying: First, conventional drying to a moisture content of ∼10% at 60 and 80 °C. Then, vacuum drying at 60 °C and 65 mbar pressure. Drying time: 3 h at 80 °C + vacuum and 6 h at 60 °C + vacuum. Final moisture: ∼3–4%.	• TPC was significantly affected by drying conditions, with higher values generally observed at 80 °C compared with 60 °C; the combination of atmospheric and vacuum drying at 80 °C yielded the highest TPC. • Antioxidant activity (FRAP) was strongly influenced by drying temperature and atmosphere, with the highest values obtained at 60 °C under atmospheric conditions and marked reductions at 80 °C. • TPC and antioxidant activity did not follow the same trend, indicating that higher phenolic concentration did not necessarily translate into higher reducing power, likely due to drying-induced structural changes in phenolic compounds.	Gonelimali et al. (2021)
Portugal	Hot-air drying. Temperature: from 40 to 100 °C. Relative air humidity: 35 and 60%. Drying time: 3.6–9.9 h. Microwave hydro-diffusion and gravity drying (MHG): Power: 300, 400, 500, 600, and 900 W. Drying time: 0.17–0.48 h.	• MHG drastically shortened drying time (≈1.0–2.6 h) compared with hot-air drying (≈3.6–9.9 h), while processing larger sample masses and achieving higher water removal rates. • High-temperature hot-air drying (>70 °C) was associated with degradation of key phenolics (e.g., 5-caffeoylquinic acid and flavan-3-ols), whereas MHG minimized thermal damage due to rapid volumetric heating. • Phenolic compounds remained recoverable from MHG-dried pomace even after 2 years of storage, indicating long-term compositional stability. • MHG enabled simultaneous recovery of soluble phenolics and carbohydrates in the condensate fractions. • Polysaccharides (mainly pectic fractions) were largely preserved after MHG drying.	Fernandes et al. (2021)
India	Microwave-assisted vacuum drying (compared to hot air oven drying). Temperature: 50 °C and 60 °C for microwave-assisted vacuum drying and oven drying, respectively. Sample weight: 5 kg. Drying time: 3 and 24 h for vacuum-assisted microwave and oven drying, respectively.	• TPC and antioxidant activity (DPPH, FRAP) were consistently higher in microwave-assisted vacuum–dried samples than in hot-air–dried counterparts. • Hot-air oven drying resulted in greater losses of phenolics and antioxidant capacity, attributed to longer exposure to heat and oxygen. • Microwave-assisted vacuum drying substantially reduced drying time while maintaining superior bioactive retention. • Improvements in phenolic retention were directly reflected in higher antioxidant performance of the resulting powder.	Bhat et al. (2023)
USA	Vacuum belt drying (compared to freeze drying). Temperature: 80, 95 or 110 °C). Pressure: 2.9 kPa	• Vacuum-belt drying preserved TPC effectively across 80–95 °C, with values not significantly different from freeze-dried pomace; the highest retention was observed at 80 °C and 95 °C, while slight reductions occurred at 110 °C. • TAC was highest in samples dried at 80 °C and declined with increasing temperature; TAC in pomace dried at 80–95 °C was comparable to or higher than that of freeze-dried pomace.	Yan and Kerr (2013)
USA	Before drying, apple pomace samples were subjected to fermentetaion (12*h*) and ultrasonication at ultrasonication amplitude of 25, 37, or 50 μm (1–3 min). Hot air oven drying. Temperature: 50 °C. Microwave drying. Power: 90 W.	• Drying method significantly affected TPC and antioxidant activity, with microwave drying outperforming hot-air oven drying under all pretreatment conditions. • TPC and antioxidant activity increased when microwave drying was combined with fermentation and ultrasonication, relative to oven drying. • Maximum TPC and antioxidant activity were obtained under microwave drying after fermentation and ultrasonication, indicating strong synergistic effects. • Ultrasonication amplitude positively influenced TPC and antioxidant activity, whereas ultrasonication time had no significant effect. • Antioxidant activity showed a strong positive correlation with TPC, confirming phenolics as the main contributors to radical scavenging capacity.	Lohani and Muthukumarappan (2014)

#### 3.1.1 Conventional drying

Across conventional HAD regimes, apple pomace phenolic retention is governed by the interplay of temperature, oxygen exposure, and residence time, with enzyme inactivation (e.g., blanching) moderating losses (Heras-Ramírez et al., 2011). Drying at 50–80 °C reduced the TPC in both blanched and unblanched apple pomace, with the steepest declines above 70 °C; notably, caffeic and *p-*coumaric acids decreased most in unblanched material, highlighting the contribution of residual polyphenol oxidase. Similarly, air drying tends to modify the phenolic composition; although total phenolic content remains within ≈5–15 g/kg DM, a greater proportion of polymerized procyanidins is observed, indicating changes in extractability and redox characteristics rather than a uniform loss (Birtic et al., 2019). Flavonoids follow these patterns closely, with quercetin glycosides and phloridzin declining as temperature and time increase; blanching before drying consistently improves retention under otherwise identical thermal loads (Heras-Ramírez et al., 2011). Anthocyanins are the most heat-sensitive fraction under hot air, showing marked reductions once temperatures exceed ≈70–80 °C, consistent with pigment cleavage and oxidation in aerated conditions (Heras-Ramírez et al., 2011). The net effect is mirrored functionally, as antioxidant activity diminishes as phenolics and flavonoids fall, although pre-blanching partially preserves activity at a given temperature relative to unblanched controls.

Conventional drying also affects the structural and functional attributes of the pectin fraction, which is fundamental to sugar and pectin co-valorisation. While galacturonic acid content of extracted apple pectin remains ≈60% and is practically unaffected across 60–105 °C, higher temperatures substantially depress pectin molecular weight (from ∼122,000 to ∼57,000 Da) and modify degree of methoxylation, with consequences for gel strength; among the tested setpoints, ∼80 °C produced the highest gel point, whereas 105 °C impaired gel performance despite similar methoxylation, indicating thermally driven chain scission during pomace drying (Constenla et al., 2002).

#### 3.1.2 Emerging and hybrid drying techniques

Low-oxygen and rapid-transfer methods consistently mitigate the oxidative and thermal penalties seen in HAD, yielding phenolic and pigment levels that approach freeze-dried benchmarks while maintaining polysaccharide structure. VBD at 80–95 °C (≈2.9 kPa) produced pomace with 44.9–51.9 g gallic acid equivalents (GAE)/kg DM total phenolics not significantly different from freeze-dried controls; under the same conditions, TAC reached ≈74 mg cyanidin-3-glucoside equivalents (C3G)/kg DM at 80 °C, again matching lyophilised references but in a fraction of the time (Yan & Kerr, 2013). MWVD further elevated phenolic retention relative to oven drying, with TPC 5.21 ± 0.09 mg GAE/g DM versus 3.14 ± 0.06 mg GAE/g DM, and correspondingly stronger antioxidant responses DPPH (2,2-diphenyl-1-picrylhydrazyl) radical scavenging activity 93 ± 1%; Ferric Reducing Antioxidant Power (FRAP) 3.22 μg/mg vs. 89 ± 1% and 2.22 μg/mg for hot air), underscoring the synergy between volumetric heating and oxygen restriction (Bhat et al., 2023). MHG likewise shortened drying by more than half while yielding dried pomace from which phenolics remained readily recoverable after prolonged storage, indicating robust preservation under the milder thermal–oxidative load (Fernandes et al., 2021).

Similar improvements were also observed for flavonoids, as under VBD and MWVD, quercetin glycosides and phloridzin were retained at levels comparable to FD, with total flavonoids remaining within ≈9–11 mg QE/g DM, closely matching FD values of ≈10–12 mg QE/g DM (Bhat et al., 2023; Yan & Kerr, 2013). Enhanced phenolic and flavonoid retention translates directly to antioxidant capacity, with vacuum−/microwave-based methods routinely outperforming hot air in antioxidant metrics (Bhat et al., 2023). Importantly, these approaches also preserve the carbohydrate matrix that motivates sugar/pectin co-valorisation: VBD yielded total dietary fiber 442–495 g/kg DM, statistically indistinguishable from freeze-dried pomace (≈480 g/kg DM), confirming maintenance of cell-wall polysaccharides under reduced pressure (Yan & Kerr, 2013).

Taken together, HAD above ≈70 °C accelerates oxidative degradation and polymerization of phenolics with concomitant losses in antioxidant activity, whereas VBD, MWVD, and MHG consistently protect phenolics, flavonoids, and peel-derived anthocyanins while sustaining the fiber/pectin framework. Because apple pomace is simultaneously a source of antioxidants and of fermentable sugars/pectin for downstream processes, regimes combining moderate temperatures (≈60–80 °C), low oxygen, and short residence times best align with dual-purpose valorisation.

### 3.2 Grape pomace

Grape pomace is the principal solid by-product of winemaking, representing 20–30% of processed grape mass and arising mainly from skins, seeds, and residual pulp after pressing and fermentation (Lopes et al., 2025). Because 45–65% of total grape phenolics remain in the pomace, it is substantially richer in polyphenols than the corresponding wine, especially in red varieties where anthocyanins and flavonoids dominate (Yang et al., 2024). Seed fractions contain the highest levels of extractable phenolics (often 50–226 mg GAE/g DM) largely composed of flavan-3-ols, while skins are enriched in flavonol glycosides, hydroxycinnamic acid derivatives, and anthocyanins (Yang et al., 2024). These compositional attributes, together with the large global volume of pomace generated annually, position grape pomace as a major and underutilized source of bioactive compounds for value-added applications (Lopes et al., 2025). Grape pomace drying has been investigated widely, and the findings of those studies have been summarized in Table 2.

**Table 2:** Summary of studies investigated the effect of different drying techniques on the bioactive properties of grape pomace.

Grape pomace origin	Drying technique and methodology	Key findings	Reference
Canada	Convective drying (compared to freeze drying). Temperature: 50–70 °C. Load: half load (0.73 kg, 3.06 kg/m^2^) or full load (1.46 kg, 6.14 kg/m^2^). Air velocity: 0.366 m/s	• TPC remained stable across all convective drying temperatures (50–70 °C) and tray loads, showing no significant change relative to freeze-dried samples. • TAC was lower than in freeze-dried samples under all air-drying conditions, with the greatest losses occurring at full tray load, especially at 70 °C. • Antioxidant activity was generally maintained across drying temperatures and loads, with only minor reductions compared to freeze-dried and no clear temperature or load-dependent pattern.	Ross et al. (2020)
Türkiye	Heat pump drying (compared to undried sample). Temperature and time: 45 and 50 °C, ∼70 and 90 min. Air velocity: at air velocity of 1 m/s. Sample weight: 500 g	• TPC decreased after drying compared to raw pomace, with greater losses at 50 °C than at 45 °C; lower-temperature heat pump drying better preserved phenolics. • TAC was substantially reduced by drying at both temperatures, with a slightly higher degradation at 50 °C; even a 5 °C increase caused noticeable anthocyanin loss. • Antioxidant activity (DPPH) declined after drying but did not differ significantly between 45 and 50 °C, indicating general retention of radical scavenging capacity. • Individual flavan-3-ols (catechin and epicatechin) decreased with drying, with greater losses at higher temperature, showing sensitivity to thermal degradation.	Aktaş et al. (2019)
Türkiye	Heat pump drying. Temperature: 45 °C. Air velocity: 1.5, 2.0 and 2.5 m/s. Drying time: 1040, 840 and 720 min for 1.5, 2.0 and 2.5 m/s air velocity, respectively.	• TPC, TTC, and antioxidant activity declined after drying relative to fresh pomace, regardless of air velocity. • Moderate air velocity (2.0 m/s) gave the highest retention of TPC and tannins among the tested conditions. • TAC was the least affected bioactive parameter, showing only minor reductions across drying conditions. • Antioxidant activity followed the same trend as TPC, with moderate air velocity preserving activity better than low or high velocities. • Individual phenolics (catechin, epicatechin, trans-resveratrol) were highly sensitive to drying, with the greatest losses observed at the highest air velocity.	Taseri et al. (2018)
Spain	Climatic chamber drying (compared to fresh sample): drying to constant weight at 40 °C, 10% relative humidity and in darkness	• TPC, TAC, and TTC all increased markedly after drying at 40 °C and 10% RH, with TPC and TAC rising by approximately threefold and TTC by ∼1.5–2-fold relative to fresh pomace. • Antioxidant activity increased substantially following drying compared with fresh samples. • Individual phenolic families (phenolic acids, flavan-3-ols, flavonols, and anthocyanins) all increased in extractable concentration after drying, rather than decreasing.	Carmona-Jimenez et al. (2018)
Greece	Convective hot-air drying. Temperature: 60, 65, 70, 75, 80, and 85 °C. Air velocity: 1.2 m/s. Drying time: 1560, 1170, 950, 710, 420, 300 min for 60, 65, 70, 75, 80, and 85 °C, respectively.	• TPC decreased progressively with increasing convective drying temperature (60–85 °C), with retention best maintained at ≤60–65 °C. • Higher temperatures accelerated phenolic degradation, with prolonged exposure causing irreversible losses. • Anthocyanins were especially heat-sensitive, showing sharp declines at elevated temperatures (>60 °C).	Goula et al. (2016)
Spain	Air-circulating oven drying (compared to freeze drying). Temperature: 60, 100, and 140 °C. Air velocity: 2.3 m^3^/min with a tray load of 10 kg/m^2^ up to a maximum moisture content of 8.0%.	• TPC and TTC were not significantly affected when drying was conducted at 60 °C, remaining comparable to freeze-dried reference samples. • At higher temperatures (100 and 140 °C), TPC declined progressively (≈19% and ≈33% losses, respectively), indicating strong temperature sensitivity. • TTC showed greater thermal stability than TPC, with smaller relative losses at 100 and 140 °C. • Antioxidant activity remained stable at 60 °C but decreased markedly at 100 °C (≈28% loss) and 140 °C (≈50% loss). • Anthocyanins were particularly sensitive to high temperatures, with substantial degradation evident at 140 °C.	Larrauri et al. (1997)
USA	Convective hot-air drying. Temperatures: 40, 60, 103, and 125 °C (for 72, 48, 16, and 8 h, respectively).	• Procyanidins were stable at 40 °C (72 h) but declined significantly at ≥60 °C; no further losses were observed between 105 and 125 °C. • TAC was not significantly affected at 40 °C, but dropped sharply above 60 °C, with the highest loss at 125 °C (∼70% reduction).	Khanal et al. (2010)
USA	Vacuum belt drying. Temperatures: Eight different combinations ranging from 60 to 120 °C. Pressure: 3–5 kPa. Drying time: from 60 to 90 min. Convective hot-air drying. Temperatures: 70 and 80 °C. Drying time: 180 and 240 min. Air velocity: 0.2, 0.4, and 0.6 m/s. Freeze drying. Pressure: 0.5 Pa. Drying time: to reduce the moisture content to 1.2 g/100 g and 1.8 g/100 g was 14 and 16 h.	• Freeze-drying yielded the highest TPC and antioxidant activity for both 2 and 4 mm pomace discs, serving as the quality benchmark. • Vacuum belt drying preserved TPC and antioxidant activity at levels statistically comparable to freeze-drying under several optimized temperature–time profiles, while reducing drying time to less than one-quarter of freeze-drying. • Hot-air drying resulted in greater losses of TPC and antioxidant activity compared to freeze-drying and optimized VBD, particularly at longer drying times. • Thinner layers (2 mm) consistently showed better retention of TPC and antioxidant activity than thicker samples (4 mm) across all drying methods. • Antioxidant activity closely tracked changes in TPC.	Vashisth et al. (2011)
USA	Convective hot-air drying. Temperature: 40 °C. Drying time: 48 h. Sample weight: 500 g Vacuum oven drying. Pressure: 27 Pa Air drying: Temperature: 25 °C. Drying time: 72 h Freeze drying: Temperature: −55 °C. Pressure: 17.33 Pa. Drying time: 60 h	• Freeze-drying consistently retained the highest TPC, TAC, TFC, and antioxidant activity across all samples (Pinot Noir and Merlot; pomace and skins). • Conventional oven drying (40 °C), vacuum drying (40 °C), and ambient air drying (25 °C) resulted in significantly lower TPC and TAC than freeze-drying, with no major differences among these three air-based methods. • TAC losses were particularly pronounced under vacuum drying, which retained only ∼45–73% of the anthocyanin levels of freeze-dried samples, depending on variety and matrix (pomace vs skins). • Antioxidant activity followed the same trend as TPC, with freeze-dried samples showing the highest values; air-dried samples retained ∼76–87% of freeze-dried antioxidant activity, while oven- and vacuum-dried samples showed greater reductions. • TFC was highest in freeze-dried samples and generally lower under all air-based drying methods, although differences between vacuum, oven, and air drying were not always statistically significant. • Across all drying methods, pomace (skins + seeds) consistently contained higher TPC, TFC, and antioxidant activity than skins alone, while skins contained higher TAC.	Tseng and Zhao (2012)
Brazil	Convection drying (compared to fresh samples). Temperature: 40, 50, and 60 °C. Air velocity: 0.42 m/s. Drying time: 6 h.	• TPC and antioxidant activity declined slightly over drying time at all temperatures, but retention was highest at 60 °C compared with 40 and 50 °C. • Drying at 60 °C resulted in the best preservation of TPC and antioxidant activity, outperforming both 40 and 50 °C. • At 40 and 50 °C, greater losses of TPC and antioxidant activity were observed, likely due to insufficient inactivation of polyphenol oxidase. • Antioxidant activity followed the same trend as TPC. • Lower water activity at higher temperature (60 °C) was associated with improved stability of phenolics and antioxidant activity.	Teles et al. (2018)
China	Convective drying. Temperature: 60, 70, 80, and 90 °C. Sample weight: 200 g. Infrared drying. The distance from the infrared emitter to the pomace: ∼20 cm. The dryer had 12,250 W electrical emitters. Sample weight: 200 g. Sequential infrared and convective drying. Sample weight: 200 g. First, the pomace was dehydrated for 7, 14, 21, or 28; then, it was dried in a convection hot-air oven at 65 °C.	• Infrared drying resulted in the highest retention of both TPC and proanthocyanidins among all tested methods. • Convective drying caused progressive losses of TPC and proanthocyanidins as temperature increased, with the lowest retention observed at 90 °C. • At 60 °C, convective drying preserved phenolics relatively well, with no major differences compared to freeze-drying reported in prior studies. • Sequential infrared–convective drying improved retention of TPC and proanthocyanidins compared to convective drying alone, with longer IR pre-treatment further enhancing preservation.	Sui et al. (2014)
Croatia	Vacuum drying. Temperature: 35, 50 and 70 °C. Pressure: 100 mbar. Drying time: 12, 5, and 3 h for 35, 50 and 70 °C, respectively. Hot air drying. Temperature: 70 °C. Pressure: Room pressure. Drying time: 7 h. Open sun drying. Temperature: 31.99 °C. Pressure: Room pressure. Drying time: 26 h.	• TPC was largely stable across drying methods, with only minor losses under vacuum drying at 35–50 °C; highest stability was observed at 70 °C. • Tannins were more sensitive than total phenolics, with greatest degradation under conventional hot-air drying and open sun drying; best retention occurred under vacuum drying at 70 °C. • Tartaric acid showed notable sensitivity to oxygen and prolonged exposure, with the highest losses under hot-air and sun drying; vacuum drying (50–70 °C) preserved tartaric acid most effectively.	Sokac et al. (2022)
Chile	Vacuum drying: Temperature: 50, 60, 75, 90, and 100 °C. Pressure: 100 mbar Convective drying: Temperature: 40, 50, 60, 70, and 80 °C. Air velocity: 1.5 m/s. Freeze drying: Temperature: −80 °C. D time: 12 h, after this period, adjust to-50 °C at 0.125 mbar.	• TPC and TFC were strongly affected by drying method and temperature, with the highest retention observed under vacuum drying at 60 °C, reaching levels comparable to or higher than freeze-dried samples. • Both TPC and TFC declined at higher temperatures (≥75 °C) under vacuum drying and at the lowest temperature (50 °C) under convective drying, indicating that prolonged drying times were more detrimental than moderate thermal exposure. • Antioxidant activity (ORAC) peaked at 60 °C for both drying methods, with convective drying at 60 °C yielding the highest values overall; freeze-dried samples did not outperform optimally air- or vacuum-dried samples. • Very low ORAC values were observed at vacuum 50 °C and at ≥75 °C, reflecting combined effects of prolonged exposure (low temperature) or thermal degradation (high temperature). • Individual phenolics such as catechin, epicatechin, gallic acid, and rutin were best preserved under vacuum drying at 60 °C, with higher peak intensities than freeze-dried samples.	Poblete et al. (2024)

#### 3.2.1 Conventional drying

The influence of conventional drying on the phenolic composition of grape pomace has been extensively examined, with temperature and exposure time identified as the dominant parameters shaping the stability of polyphenols (Larrauri et al., 1997). Drying red grape pomace peels at 60, 100, and 140 °C under an airflow of 2.3 m^3^/min and a tray load of 10 kg/m^2^ resulted in progressive degradation of extractable polyphenols. Compared to freeze-dried controls, total extractable polyphenols decreased by 18.6% and 32.6% at 100 °C and 140 °C, respectively, while total tannin content (TTC) declined by 11.1% and 16.6%. Antioxidant activity was also substantially reduced (−28% at 100 °C and − 50% at 140 °C), confirming that high temperatures accelerate oxidative and polymerization reactions detrimental to phenolic stability (Larrauri et al., 1997). At lower temperatures, convective air drying between 50 and 70 °C can retain TPC effectively when airflow and bed depth are optimized (Ross et al., 2020). In high- and low-load trays, TPC ranged between 3421 and 3965 mg GAE/100 g DM for full loads and 3561–3965 mg GAE/100 g DM for half loads, compared to 4236 mg GAE/100 g DM in freeze-dried samples. Although overall phenolic levels were similar, composition shifted toward lower-molecular-weight phenols, suggesting partial degradation of polymeric fractions. Low-temperature drying can also enhance phenolic extractability. Controlled-humidity drying of red grape pomace at 40 °C and 10% relative humidity increased TPC by approximately 3-fold (from 14.7 to 45.8 mg GAE/g DM) and similarly elevated TAC and TTC, likely through improved mass transfer and release of bound phenolics from the cell wall matrix (Carmona-Jimenez et al., 2018). These findings suggest that mild thermal stress can increase extractable phenolics even as absolute contents remain thermally constrained.

Tannins are notably heat-sensitive, exhibiting proportional losses with increasing temperature. Under the same drying conditions described by Larrauri et al. (1997), condensed tannins declined more sharply than total phenolics, consistent with depolymerization and oxidative condensation during extended heating. Reducing residence time to below 6 h mitigated tannin degradation, indicating that time–temperature interactions are as critical as absolute setpoint (Carmona-Jimenez et al., 2018). The behavior of flavonoids, especially catechin, epicatechin, and procyanidins, follows similar kinetics. Airflow modulation experiments revealed that drying grape pomace at 45 °C with varying air velocities (1.5, 2.0, and 2.5 m/s) significantly altered TPC and tannin concentrations (Taseri et al., 2018). The highest values were achieved at 2.0 m/s (TPC: 38.1 mg GAE/g DM; TTC: 45.1 mg tannic acid equivalents (TAE)/g DM), indicating an optimal mass-transfer regime where enhanced convection improves drying kinetics without causing oxidative damage. Extended heating experiments further clarify the degradation pattern of flavan-3-ols. Heating freeze-dried grape pomace at 40–125 °C for 8–72 h produced steady declines in procyanidin concentrations from 60 °C upward, with minimal further loss beyond 105 °C (Khanal et al., 2010). This plateau suggests that phenolic polymer degradation becomes diffusion-limited at high temperature, leading to partial stabilization after the initial breakdown phase (Khanal et al., 2010).

Anthocyanins display high thermal lability, too. Significant red-color fading and anthocyanin losses were reported above 100 °C, whereas drying at 60 °C largely preserved pigment integrity (Larrauri et al., 1997). Under prolonged drying at 80 °C, TAC declined by 45–60%, whereas at 40 °C, extractability rather than degradation dominated, leading to higher measurable anthocyanin values (Carmona-Jimenez et al., 2018). Phenolic acids and stilbenes show comparatively greater thermal resilience. Drying grape pomace at 40 °C increased concentrations of protocatechuic, syringic, and vanillic acids (to as high as 599 mg/kg depending on variety) due to cleavage of ester-linked forms. In muscadine pomace, resveratrol decreased by only ∼17% following air drying at 70 °C, reflecting the compound's inherent stability under moderate heat (Vashisth et al., 2011).

Antioxidant capacity tracked the degradation of phenolics but was partially compensated by the generation of phenolic acid derivatives with redox activity. Under air drying at 70 °C, antioxidant values declined by approximately 10–20% compared to freeze-dried controls, while low-humidity drying at 40 °C significantly enhanced antioxidant readings relative to fresh samples (Carmona-Jimenez et al., 2018; Ross et al., 2020).

Beyond compositional changes, several studies on grape pomace have also examined the drying process from a kinetic and process-engineering perspective, allowing a more mechanistic interpretation of quality retention. Convective drying of grape pomace has been successfully described using diffusion-based and empirical thin-layer models, with effective moisture diffusivity increasing as temperature rises and drying time decreases (Goula et al., 2016; Poblete et al., 2024). Goula et al. (2016) demonstrated that the combined effect of temperature and moisture content could be expressed through an empirical diffusivity relationship, while the Midilli model provided the best fit to the drying curves over 60–85 °C, confirming the strong temperature dependence of internal mass transfer. Similarly, Poblete et al. (2024) showed that convective drying kinetics of grape pomace could be adequately captured using Fick-based and Midilli–Kucuk approaches, supporting the use of these models for process optimization.

Overall, evidence across temperature–time–load conditions indicates that ≤60–70 °C combined with reduced bed depth maintains phenolic integrity while limiting subclass losses. Half-load trays ensure better air distribution and higher antioxidant retention than full-load configurations (Ross et al., 2020). Low-temperature, low-humidity drying (≈40 °C) can enhance extractability of phenolics, tannins, and anthocyanins relative to fresh matrices (Carmona-Jimenez et al., 2018). Air velocity could be maintained around 2.0 m/s to balance drying kinetics and oxidation risk (Taseri et al., 2018). Extended exposure above 60 °C needs to be avoided to prevent procyanidin degradation (Khanal et al., 2010). Excessive temperatures (>100 °C) cause pigment fading and tannin loss, whereas ∼60 °C stabilizes both color and phenolics during convective processing (Larrauri et al., 1997). Vacuum-assisted conditions outperform conventional air drying when oxygen sensitivity is a limiting factor (Vashisth et al., 2011).

#### 3.2.2 Emerging and hybrid drying techniques

Advances in drying technology have sought to mitigate oxidative losses through vacuum, IR, MD, and RW systems, each aiming to shorten processing times while preserving the phenolic matrix. In *Vitis vinifera* “Moscatel” pomace, vacuum drying at 60 °C and 100 mbar yielded the highest TPC (28 mg GAE/g DM) and TFC (13 mg QE/g DM) while reducing drying time to ∼210 min, outperforming convective runs at the same temperature by limiting oxygen exposure (Poblete et al., 2024). The same study identified gallic and 4-hydroxybenzoic acids, catechin, epicatechin, and rutin as the major retained compounds after vacuum drying.

IR-assisted drying also improved the preservation of flavan-3-ols. At 70 °C, IR-dried grape pomace retained up to 90% of catechin and 85% of epicatechin relative to freeze-dried samples, while convective drying at 80 °C caused over 40% losses, showing that short, surface-targeted heating under IR reduces flavonoid oxidation (Taseri et al., 2018). Rapid drying kinetics and limited oxygen exposure are the main advantages of this approach. RW drying further enhanced anthocyanin and total phenolic retention. Drying red grape pomace at 70 °C in a thin film preserved approximately 78% of TAC and 85% of TPC compared to freeze-dried controls, while significantly shortening drying time (Sokac et al., 2022). These findings highlight the importance of uniform heat transfer and reduced exposure time for anthocyanin stabilization.

MD also demonstrated improved retention relative to conventional heating. Under conditions delivering equivalent water removal, MD maintained ∼88% of TAC and reduced process time by 50%, indicating that volumetric energy input promotes rapid internal heating with minimal oxidative degradation (Aktaş et al., 2019). Hybrid approaches such as combining IR or microwave pre-treatments with convective finishes have also been proposed to enhance efficiency while maintaining phenolic stability. Sequential drying at ∼65 °C combining infrared and convective stages shortened total processing time while reducing total phenolic losses compared with convective drying alone (Sui et al., 2014). Similarly, convective–vacuum combinations preserved tannins and phenolic acids more effectively than single-stage convective processes (Teles et al., 2018).

Under all hybrid configurations, anthocyanin retention was consistently higher than in conventional drying, and phenolic acids such as gallic, protocatechuic, syringic, and vanillic acids remained largely unaffected by mild vacuum or IR exposure (Carmona-Jimenez et al., 2018). Stilbenes, particularly resveratrol, also exhibited strong thermal resilience under IR and microwave-assisted systems (Vashisth et al., 2011). The antioxidant activity of grape pomace dried under vacuum or hybrid conditions mirrored the phenolic preservation trends. Vacuum drying at 60 °C yielded antioxidant values comparable to freeze-dried samples, while RW and microwave-assisted systems maintained 90–95% of antioxidant capacity relative to lyophilized controls (Aktaş et al., 2019; Poblete et al., 2024). These results collectively confirm that oxygen limitation and time compression are decisive factors in retaining the redox-active constituents of grape pomace.

In contrast to conventional systems, emerging grape pomace drying technologies have increasingly been evaluated not only for bioactive retention, but also for drying kinetics, energy performance, and industrial feasibility. VD of grape pomace showed the highest diffusivity and shortest effective drying time at 60 °C, with the Midilli–Kucuk model again providing the best fit, indicating that moderate-temperature vacuum conditions favor both rapid moisture removal and polyphenol preservation (Poblete et al., 2024). Likewise, Sokac et al. (2022) applied Peleg's model and thermodynamic analysis to VD of grape pomace, showing that higher drying temperature accelerated desorption and reduced the energy required to remove bound water, while activation energy and Gibbs free energy values further characterized the non-spontaneous and temperature-dependent nature of the process. Heat-pump-assisted systems also offered relevant energy advantages. For instance, Taseri et al. (2018) reported that heat-pump drying reduced energy consumption by up to 51% relative to the convective control, whereas Aktaş et al. (2019) showed that closed-loop heat-pump drying achieved coefficient of performance values above 3 and could be recommended as an industrial alternative when low-temperature drying is required.

Taken together, VD at ∼60 °C and 100 mbar provides the best compromise between bioactive preservation and process efficiency, yielding superior retention of phenolics, flavonoids, and antioxidant activity (Poblete et al., 2024). IR-assisted drying at ∼70 °C effectively preserves catechin and epicatechin by accelerating surface evaporation and reducing oxidative exposure (Taseri et al., 2018). RW drying at ∼70 °C offers exceptional anthocyanin protection, retaining ∼78% of total monomeric anthocyanins while reducing energy use (Sokac et al., 2022). MD maintains high TAC, retaining ∼88% of monomeric anthocyanins while halving drying time relative to convective drying (Aktaş et al., 2019). Sequential or hybrid methods that combine moderate pre-drying (≤60 °C) with a rapid IR or microwave finish balance moisture removal with minimal anthocyanin degradation (Sui et al., 2014; Teles et al., 2018).

### 3.3 Blueberry pomace

Blueberries (*Vaccinium* sect. *Cyanococcus*), which are native to North America, have four main species: rabbit-eye (*V. virgatum*), lowbush (*V. angustifolium*), northern highbush (*V. corymbosum*), and southern highbush (*V. formosum*) (Huang et al., 2025). Blueberry pomace has been mainly investigated for its polyphenolic content, particularly anthocyanins, rather than fiber. Although the total phenolic and anthocyanin content of fresh blueberry pomace is not well documented, it may contain approximately 34–37 mg GAE/g FM and 9.5–14 mg C3G/g FM, respectively (Zhang et al., 2023; Zielinska & Michalska, 2018). It is known that blueberry cultivar, growing conditions, maturity stage, processing method (e.g., juicing), and chemical analysis method can all affect the bioactive content (Ashique et al., 2024). The total phenolic content of dried pomace can also vary widely, ranging from 5.9 mg GAE/g DM to as high as 217 mg GAE/100 g DM, depending mainly on the blueberry sample and drying method. Similarly, the total monomeric anthocyanin content can range between 0.1 and 19.6 mg C3G/g DM (Zhang et al., 2023; Zhang et al., 2025). The key polyphenolic compounds present in blueberry pomace include delphinidin-3-galactoside (4.32 mg/g DM), malvidin-3-arabinoside (4.54 mg/g DM), and delphinidin-3-glucoside (4.62 mg/g DM) (Zhang et al., 2025). Blueberry pomace is also one of those fruit pomaces that has been investigated by many researchers, the findings of those studies have been summarized in Table 3.

**Table 3:** Summary of studies investigated the effect of different drying techniques on the bioactive properties of different berry pomaces.

Fruit pomace	Pomace origin	Drying technique and methodology	Key findings	Reference
Blueberry	Spain	Air drying (compared to fresh sample). Temperature and time: 60 °C for ∼270 min and 70 °C for 210 min.	• TPC was moderately affected by drying temperature: drying at 60 °C caused a small decrease (∼10%), while drying at 70 °C showed no significant loss compared to fresh pomace. However, milling (especially fine milling) substantially reduced TPC due to increased oxygen exposure. • TAC was strongly temperature- and process-dependent: ∼75% retention at 60 °C and ∼ 66% retention at 70 °C were achieved, indicating good thermal stability in the pomace matrix. Coarse milling did not further reduce TAC, but fine milling caused a significant additional decline. • Antioxidant activity (DPPH) followed the same trend as TAC, decreasing with higher drying temperature and further declining with fine milling; strong correlation was observed between DPPH values and anthocyanin content. • •Antioxidant activity (ABTS) showed larger losses (≈31–37%) compared to DPPH, with the best retention observed at 70 °C without milling; milling did not markedly worsen ABTS activity beyond the losses already induced by drying.	Calabuig-Jiménez et al. (2022)
	Mexico	Hot air convective drying (compared to freeze drying). Temperature: 50, 60, 70, 80, and 90 °C. Air velocity: 2.5 m/s. Drying time: 270 (50 °C) to 1080 min (90 °C).	• TPC followed a non-linear trend with temperature, decreasing at 50–60 °C but peaking at 70 °C, where it reached the highest value among all convective treatments; higher temperatures (80–90 °C) showed slightly lower but still elevated TPC compared to low-temperature drying. • TAC decreased progressively with increasing drying temperature, showing substantial losses relative to freeze-dried samples, with the greatest degradation observed at 80–90 °C. • Antioxidant activity (DPPH and ABTS) was lowest at 50–60 °C but increased significantly at 70–90 °C, mirroring the trend in TPC and suggesting enhanced release of bound phenolics at higher temperatures. • The optimal compromise between TPC retention, antioxidant activity, and process efficiency was observed at 70 °C, which combined high bioactive retention with reduced drying time and energy consumption.	José P. Tejeda-Miramontes et al. (2024)
	China	Hot air convective drying. Temperature: 40, 50, 60, and 70 °C. Air velocity: 0.1 m/s. Freeze frying. Temperature: 49 °C. Pressure: 14 bar. Drying time: 36 h. Microwave vacuum drying. Temperature: 60 °C. Power: 7.5 W/g, Pressure: −0.09 Pa. Drying time: 1 h. Microwave freeze vacuum drying. Temperature: −49 °C. Power: 400 W. Vacuum degree: −0.08 kPa. Drying time: 4 h.	• TPC was highest under microwave vacuum drying (MVD), followed by microwave freeze vacuum drying (MFD) and freeze-drying (FD), while all hot-air drying (HAD) treatments showed substantially lower TPC; losses increased with increasing air temperature. • TAC followed the same trend as TPC, with MVD showing the highest retention, followed by MFD and FD; HAD caused severe anthocyanin degradation, particularly at 60–70 °C. • Antioxidant activity (ABTS and DPPH) was significantly higher in MVD-, MFD-, and FD-treated samples compared with HAD; MVD consistently produced the strongest radical scavenging activity. • Individual anthocyanins were better preserved under MVD, MFD, and FD, while HAD caused extensive degradation of most monomeric anthocyanins, with higher temperatures leading to near-complete loss of several compounds.	Zhang et al. (2023)
	China	Hot air convective drying.. Temperature: 50, 60, and 80 °C. Drying time: 24, 16, and 8 h for 50, 60, and 80 °C, respectively. Microwave-assisted hot air drying. Power: 70 W. Drying time n: 30 min. Vacuum freeze drying. Temperature: −49 °C. Pressure: 14 bar. Drying time: 48 h	• TPC and TAC were highest in vacuum freeze-dried (VFD) pomace, followed by HAD at 50 °C and 60 °C; both parameters declined markedly at 80 °C, indicating strong thermal sensitivity. • Microwave-assisted hot air drying (MHAD) shortened drying time by ∼62.5% compared with HAD at 60 °C, but resulted in lower TAC and slightly reduced TPC, highlighting a trade-off between efficiency and bioactive retention. • Antioxidant activity (DPPH and FRAP) closely mirrored trends in TPC and TAC, with the highest values in VFD samples, followed by HAD at 50–60 °C; HAD at 80 °C produced the lowest antioxidant capacity. • Individual anthocyanins (14 monomers identified) were best preserved under VFD, moderately retained under HAD at 50–60 °C, and severely degraded at 80 °C; MHAD caused additional losses compared with HAD at 60 °C. • Overall, VFD provided the best preservation of phenolics, anthocyanins, and antioxidant activity, while HAD at 50–60 °C represented the best compromise between quality retention and process efficiency.	Zhang et al. (2025)
	Poland	Hot air convective drying. Temperature: 60 and 90 °C. Microwave-vacuum drying (MWVD). Power: 1.3 W/g in the vacuum chamber rotated at 6 rpm (4–6 kPa). Combined drying (HAD + MWVD). Temperature: 60 and 90 °C.	• Drying caused substantial losses of TPC (39–76%), TAC (21–77%), and antioxidant activity (24–75%) across all methods. • Microwave–vacuum drying (MWVD) preserved TPC, TAC, and antioxidant activity best, with values comparable to non-dried pomace. • HAD at 60 °C caused the greatest degradation of all bioactive parameters; losses were smaller at 90 °C due to shorter exposure time. • Combined HAD (60 °C) + MWVD significantly improved retention of TPC, TAC, and antioxidant activity compared with HAD alone, while reducing drying time by ∼75%. • Combined HAD (90 °C) + MWVD shortened drying time but did not improve retention compared with HAD at 90 °C. • Antioxidant activity closely followed trends in both TPC and TAC, showing strong positive correlations with both parameters.	Zielinska and Michalska (2018)
	Canada - Highbush blueberry	Hot air convective drying (compared to freeze drying). Temperature: 50–70 °C. Load: half load (0.73 kg, 3.06 kg/m^2^) or full load (1.46 kg, 6.14 kg/m^2^). Air velocity: 0.366 m/s	• TPC remained stable across 50–70 °C and half/full tray loads, with no significant differences relative to freeze-dried samples. • TAC was consistently lower than in freeze-dried samples under all convective drying conditions. Losses increased with higher temperatures and with full tray load (half load retained more). • Antioxidant activity was generally maintained across temperatures and loads, remaining comparable to freeze-dried samples except at 70 °C full load, where both assays (ABTS and FRAP) showed reduced activity.	Ross et al. (2020)
	Canada - Wild lowbush blueberry	Hot air convective drying (compared to freeze drying). Temperature: 50–70 °C. Load: half load (0.73 kg, 3.06 kg/m^2^) or full load (1.46 kg, 6.14 kg/m^2^). Air velocity: 0.366 m/s	• TPC was unchanged across all convective drying temperatures (50–70 °C) and both tray loads, remaining similar to freeze-dried levels. • TAC was consistently lower than freeze-dried under all air-drying conditions. Losses increased with full tray load and at lower retention under higher temperatures (full load consistently lowest). • ABTS and FRAP values were slightly reduced relative to freeze-dried but did not differ significantly across temperatures or between half/full loads; overall antioxidant activity was broadly maintained.	Ross et al. (2020)
	USA	Hot air convective drying. Temperatures: 40, 60, 103, and 125 °C (for 72, 48, 16, and 8 h, respectively).	• Procyanidins remained stable at 40 °C (72 h) but declined significantly at ≥60 °C, with no additional losses between 105 and 125 °C. • TAC showed minimal loss at 40 °C but decreased strongly above 60 °C, with ∼52% loss at 125 °C.	Khanal et al. (2010)
Cranberry	Canada	Hot air convective drying (compared to freeze drying). Temperature: 50–70 °C. Load: half load (0.73 kg, 3.06 kg/m^2^) or full load (1.46 kg, 6.14 kg/m^2^). Air velocity: 0.366 m/s	• TPC was unchanged across 50–70 °C and half/full tray loads, showing no significant differences from freeze-dried cranberry pomace. • TAC was consistently lower than freeze-dried at all air-drying temperatures and loads, with greater losses at full tray load; highest retention occurred at 50–60 °C half load. • ABTS and FRAP values were broadly maintained across temperatures and loads, with only slight decreases relative to freeze-dried material and no strong temperature-dependent trend.	Ross et al. (2020)
Blackcurrant	Poland	Freeze drying. Pressure: 65 Pa. Drying time: 24 h. Sample weight: 500 g. Hot air convective drying. Temperature: 50, 60, 70, 80, and 90 °C. Air velocity: 0.8 m/s. Sample weght: 100 g. Microwave drying. Power: 120, 240, 360, and 480 W at 4–6 kPa. Combination of these drying method: First, blackcurrant pomace was convective pre-dried at 50, 60, 70, 80, and 90 °C to a moisture content of ∼0.25 kg/kg. This was followed by microwave finish-drying at 480 W to moisture content of 0.065 kg/kg.	• Drying caused substantial losses of TPC and antioxidant activity relative to fresh pomace across all methods. • Convective drying (50–90 °C) showed a clear temperature-dependent decline in TPC, with the highest retention at 50 °C and the lowest at 90 °C. • Antioxidant activity decreased progressively with increasing convective drying temperature; values at 90 °C were ∼ 32% lower than at 50 °C. • Microwave vacuum drying better preserved TPC and antioxidant activity than convective drying, with no clear temperature-dependent degradation trend. • Freeze-drying still caused notable reductions in both TPC and antioxidant activity compared with fresh pomace, indicating that sublimation alone did not fully prevent losses. • Combined convective–microwave vacuum drying improved retention of TPC and antioxidant activity compared with convective drying alone, while substantially shortening drying time. • Antioxidant activity closely followed TPC trends.	Michalska et al. (2017)
Raspberry	Mexico	Hot air convective drying (compared to freeze drying). Temperature: 50, 60, 70, 80, and 90 °C. Air velocity: 2.5 m/s.	• The lowest TPC and antioxidant activity was recorded at 50 °C while the highest TPC and antioxidant activity at 70 °C was obtained. • Similarly, TAC of the pomaces dried at 70 °C was the higher than those of the dried samples at the other temperatures. • Drying at 70 °C provided the optimal drying for producing high-quality dried pomace.	J. P. Tejeda-Miramontes et al. (2024)
Bignay	Philippines	Hot air convective drying (compared to fresh samples). Temperature: 45 °C. Drying time: 48 h. Freeze-drying (compared to fresh samples). Temperature: −30 °C. Pressure: 100–300 Pa. Drying time: 30 h.	• TPC decreased after both drying treatments, with freeze-drying retaining substantially higher levels than convection oven-drying (minimal loss under freeze-drying vs. ∼35% loss under oven-drying). • TAC increased after drying, particularly under freeze-drying, which led to a ∼ 176% increase relative to fresh pomace; convection oven-drying showed a smaller but still significant increase (∼23%). • TFC and condensed tannins were not significantly affected by either drying method. • Antioxidant activity was highest in freeze-dried samples across all assays (DPPH, ABTS, FRAP), while convection oven-drying led to slight reductions or no improvement compared to fresh pomace.	Zubia et al. (2023)

#### 3.3.1 Conventional drying

The impact of convective hot-air drying on the preservation of bioactive compounds in blueberry pomace has been widely investigated, revealing that moderate processing conditions can balance efficiency and bioactive retention (José P. Tejeda-Miramontes et al., 2024a). At an air velocity of 2.5 m/s and temperatures between 50 and 90 °C, TPC increased up to 13.65 ± 0.12 mg GAE/g DM at 70 °C but declined slightly at higher temperatures, indicating that moderate heat enhances phenolic extractability through cellular softening, whereas excessive exposure accelerates oxidation and polymerization (José P. Tejeda-Miramontes et al., 2024b). Consistent trends were observed in studies using cabinet dryers operating at 50–70 °C, where phenolic levels in air-dried pomace (≈1910–2050 mg GAE/100 g DM) remained statistically comparable to freeze-dried controls (Ross et al., 2020). These results collectively demonstrate that within controlled airflows, moderate heating can achieve high phenolic retention, provided that drying time and bed depth are optimized to prevent localized overheating.

Anthocyanins, being more thermolabile, exhibited greater susceptibility to degradation under convective drying. In both highbush and lowbush blueberry pomace, anthocyanin concentrations decreased by approximately 15–25% compared to freeze-dried counterparts, with losses rising sharply at higher tray loads (Ross et al., 2020). Half-load trays at 70 °C preserved up to 1360 mg C3G/100 g DM compared to 1112 mg/100 g DM in full-load trays, underscoring the critical role of airflow uniformity and reduced material thickness in limiting pigment breakdown. These observations highlight that anthocyanin retention during hot-air drying depends less on small temperature increments and more on effective moisture removal kinetics and oxygen exposure.

Despite measurable pigment degradation, the antioxidant potential of convectively dried pomace remained remarkably stable. 2,2′-Azino-bis(3-ethylbenzothiazoline-6-sulfonic acid) (ABTS) radical-scavenging activity values of 13,600–14,300 μmol Trolox equivalent (TE)/100 g DM were recorded in air-dried pomace compared to 14,628 μmol TE/100 g DM in freeze-dried samples, indicating that other polyphenols, such as phenolic acids and flavonols, may compensate for anthocyanin losses (Ross et al., 2020). This finding is corroborated by Calabuig-Jiménez et al. (2022), who reported that drying at 60–70 °C maintained over two-thirds of the anthocyanins and preserved DPPH/ABTS activities even after 20 weeks of storage. Therefore, moderate HAD can preserve functional antioxidant capacity when applied under optimized airflow and load conditions.

In terms of dietary fiber, convective drying resulted in minimal compositional disruption, supporting its use for generating fiber-rich ingredients. At 2.5 m/s airflow, total dietary fiber remained constant at approximately 20.85 g/100 g DM across 50–90 °C, with insoluble fiber slightly decreasing from 17.4 to 16.1 g/100 g DM and soluble fiber rising from 3.1 to 4.7 g/100 g DM between 50 and 80 °C (José P. Tejeda-Miramontes et al., 2024). This increase in soluble fiber likely results from the partial depolymerization of hemicellulosic structures, enhancing solubility and potential prebiotic functionality.

In addition to compositional retention, studies on blueberry pomace have examined the drying process through kinetic and transport-based analyses, providing a more quantitative basis for interpreting quality changes during convective dehydration. In cabinet and convection systems, drying behavior has been successfully described using thin-layer models, with the Page model consistently providing the best fit to experimental moisture-ratio data across temperature ranges relevant to pomace stabilization (Ross et al., 2020; José P. Tejeda-Miramontes et al., 2024). Effective moisture diffusivity increased with temperature, confirming that internal mass transfer is strongly temperature dependent, while activation energy values further quantified the sensitivity of water removal to thermal input. In José P. Tejeda-Miramontes et al. (2024), diffusivity increased from 4.00 × 10^−8^ to 2.17 × 10^−7^ m^2^/s between 50 and 90 °C, with an activation energy of 39.55 kJ/mol, whereas Ross et al. (2020) similarly showed that shorter drying times at half-load and 70 °C improved retention of blueberry bioactives relative to longer convective runs. These studies indicate that, in blueberry pomace, moderate-temperature convective drying can be interpreted not only in terms of phenolic retention, but also through diffusion-controlled moisture transport and model-based optimization. Overall, the available evidence indicates that convective drying at around 70 °C, under moderate air velocity and reduced tray load, represents a practical and scalable approach for producing dried blueberry pomace with balanced phenolic preservation, antioxidant potential, and improved soluble fiber functionality.

#### 3.3.2 Emerging and hybrid drying techniques

Emerging drying technologies integrating microwave or vacuum energy inputs have demonstrated notable improvements in the retention of phenolic compounds, anthocyanins, and antioxidant activity compared to conventional convective drying. MWVD for example, achieved the highest total phenolic (14.49 mg/g DM) and anthocyanin levels among the four tested methods (MWVD > microwave freeze drying (MFD) > vacuum freeze drying (VFD) > HAD), significantly outperforming hot-air drying at 70 °C, which yielded only 5.88 mg/g DM (Zhang et al., 2023). The superior preservation achieved by MWVD can be attributed to rapid, volumetric heating under low-oxygen vacuum, which accelerates moisture diffusion while limiting oxidative degradation. Similarly, combining convective pre-drying at 60 °C with a microwave-vacuum phase (hot-air combined drying (HACD) + MWVD) shortened total drying time by up to 91% while reducing anthocyanin losses from approximately 77% under conventional conditions to only 22%, demonstrating the potential of hybrid systems for optimizing both energy efficiency and product quality (Zielinska & Michalska, 2018).

Microwave-assisted hot-air drying (MHAD), which couples microwave energy (70 W) with subsequent hot-air exposure (60 °C), has shown to offer substantial time savings (up to 62.5% shorter cycles) yet compromises antioxidant integrity due to uneven temperature distribution and residual oxygen exposure (Zhang et al., 2025) . In contrast, VFD retains the highest phenolic and anthocyanin levels due to its low-temperature, oxygen-free conditions, though its energy and time demands remain significant. These observations suggest that microwave-vacuum and hybrid microwave-assisted processes may serve as effective middle grounds, delivering superior bioactive preservation with manageable energy costs.

The preservation of antioxidant activity across these emerging methods closely parallels the phenolic and anthocyanin trends. Both MWVD and MFD retained significantly higher DPPH and ABTS radical-scavenging activities than HAD and MHAD, whereas VFD samples consistently exhibited the strongest antioxidant profiles (Zhang et al., 2023; Zhang et al., 2025). Importantly, time–temperature interactions appear to be decisive: extended thermal exposure above 60 °C or beyond 8 h resulted in more than 50% loss of anthocyanins and procyanidins (Khanal et al., 2010). This reinforces the importance of rapid moisture removal under controlled heating flux rather than simply minimizing absolute temperature.

For blueberry pomace, the value of emerging and hybrid drying systems is reinforced by process-oriented evidence showing that improvements in bioactive retention are closely linked to accelerated moisture transport and shorter exposure times. Zielinska and Michalska (2018) showed that MWVD markedly accelerated dehydration, reducing drying time by 91% relative to hot-air convective drying, while combined HACD + MWVD reduced the convective stage by 68–75% depending on the pre-drying temperature. These differences were accompanied by substantially higher effective moisture diffusivity under microwave-assisted conditions, consistent with enhanced internal water transport under volumetric heating and reduced-pressure environments. From a practical perspective, such time compression is highly relevant because it simultaneously reduces oxygen exposure and improves process efficiency. Complementary evidence from José P. Tejeda-Miramontes et al. (2024) further highlights the importance of integrating process engineering into quality assessment as under optimized convective drying, energy consumption decreased from 13.88 to 6.95 kWh and production costs were reduced by 47%, while CO₂ emissions also declined. Overall, the collective evidence indicates that vacuum- and microwave-based drying systems, particularly MWVD and hybrid HACD + MWVD configurations, offer the most promising routes for retaining phenolics and anthocyanins while minimizing processing time and energy use. However, from a scalability and economic standpoint, well-optimized convective drying remains the most feasible industrial approach.

### 3.4 Miscellaneous berry pomaces

#### 3.4.1 Elderberry pomace

Elderberry (*Sambucus nigra*) pomace accounts for 25–40% of the fresh berries and is rich in cyanidin-3-sambubioside, cyanidin-3-glucoside, cyanidin-3-galactoside, and rutin (Mutavski et al., 2024). It has been reported that the phenolic composition of elderberry pomace is strongly influenced by drying technique, with freeze-drying outperforming convective and fluidized-bed methods in preserving TPC and TAC (Table 3) (Gomez Mattson et al., 2024). Total phenolics and anthocyanins were significantly greater in freeze-dried compared with air-dried and fluidized-bed-dried samples. Cyanidin-3-glucoside and rutin were the predominant compounds, with cyanidin-3-glucoside reaching 1139 ± 86 mg/100 g wet basis in freeze-dried pomace, compared with 661 ± 36 mg/100 g and 863 ± 163 mg/100 g in cabinet- and fluidized-bed-dried samples, respectively. Rutin followed the same trend, measuring 155 ± 7 mg/100 g wet basis in freeze-dried pomace, relative to 97 ± 3 mg/100 g in air-dried and 123 ± 23 mg/100 g in fluidized-bed-dried samples. Although both air-based methods achieved rapid dehydration (1 h for fluidized-bed; 6 h for hot air at 60 °C), this efficiency came at the cost of lower phenolic preservation. These findings reinforce the thermal sensitivity of elderberry anthocyanins, particularly cyanidin glycosides, which undergo structural degradation through glycosidic cleavage and oxidative reactions under elevated temperatures.

#### 3.4.2 Cranberry pomace

Cranberry (*Vaccinium macrocarpon*) pomace contains a broad range of polyphenols, including anthocyanins, quercetin and its glycosides, and A-type proanthocyanidins spanning degrees of polymerization 3–15 (Roopchand et al., 2013). Cranberry pomace has demonstrated high stability of total phenolics and flavonols during convective drying between 50 and 70 °C (Table 3) (Ross et al., 2020). TPC ranged from 1355 to 1517 mg GAE/100 g DM, comparable to freeze-dried controls, while flavonols were similarly stable at 286–303 mg QE/100 g DM. Anthocyanins displayed modest sensitivity to drying load: half-load drying retained pigment content (∼120 mg C3G/100 g DM) similar to freeze-dried pomace (∼147 mg C3G/100 g DM), whereas full-load at 70 °C resulted in a more notable reduction (∼107 mg C3G/100 g DM). Antioxidant capacity remained largely unaffected, with ABTS 8933–9610 μmol TE/100 g DM and FRAP 6717–7081 μmol TE/100 g DM, regardless of temperature or load. These findings suggest cranberry pomace possesses higher thermal resilience than most anthocyanin-rich matrices, and that tray load; affecting airflow and moisture gradients, plays a more critical role than temperature in retaining pigment and redox quality.

#### 3.4.3 Raspberry pomace

Red raspberry (*Rubus idaeus*) pomace is also a rich source of polyphenols, with cyanidin-3-glucoside (72.9 ± 0.1 mg/g DM) as the main anthocyanin, and chlorogenic acid (43.9 ± 0.2 mg/g DM) and protocatechuic acid (29.1 ± 0.1 mg/g DM) as the main phenolic acids (Vipotnik et al., 2025). Raspberry pomace showed strong compositional and functional stability under moderate convective drying (Table 3). Across 50–90 °C (air velocity 2.5 m/s; 10 mm layer), 70 °C emerged as the optimal balance between drying time and quality, achieving TPC 32.10 mg GAE/g DM and TAC 25.84 mg C3G/g DM (J. P. Tejeda-Miramontes et al.,2024a). In contrast, lower temperatures (50–60 °C) produced slightly higher retention than 80–90 °C but required substantially longer drying times, whereas temperatures ≥80 °C showed marked declines in both TPC and TAC, consistent with enhanced anthocyanin thermal degradation and polymerization at higher temperatures. Antioxidant capacity under these conditions was high, with DPPH 33.29 mg ascorbic acid equivalents (AAE)/g DM, ABTS 35.85 mg AAE/g DM, and half-maximal inhibitory concentration (IC_50_) values of 0.016 mg/mL (DPPH) and 0.029 mg/mL (ABTS). In addition, raspberry pomace retained substantial amounts of dietary fiber, with total dietary fiber 52.52–64.76 g/100 g DM and soluble dietary fiber increasing by 43.40% under optimized drying. Process modeling further supported the selection of 70 °C as the optimal condition, with the Page model providing the best fit to the drying curves and with effective moisture diffusivity increasing progressively with temperature. At 70 °C, the combination of a high drying rate, favorable moisture transport, and improved soluble fiber formation indicates that quality retention in raspberry pomace is closely linked to both diffusion-controlled dehydration and thermal modification of the polysaccharide matrix.

#### 3.4.4 Blackcurrant pomace

Blackcurrant (*Ribes nigrum* L.) pomace retains a diverse phenolic profile dominated by flavan-3-ols and phenolic acids, with catechin (≈0.25 mg/g DM) and epicatechin (≈0.15 mg/g DM) as the major flavonoids and hydroxybenzoic acids (≈0.49 mg/g DM) as the predominant phenolic acid fraction (Untea et al., 2024). The impact of drying technique on the total phenolic content and antioxidant capacity of blackcurrant pomace was systematically evaluated by Michalska et al. (2017). Convective drying at increasing temperatures (50–90 °C) resulted in a temperature-dependent decline in total polyphenols, with TPC decreasing from 7.1 g/kg DM at 50 °C to 3.1 g/kg DM at 90 °C, indicating substantial thermal degradation under prolonged air exposure. Freeze-drying, although often regarded as a gentle method, also led to marked losses, with antioxidant capacity approximately 60% lower than that of fresh pomace, likely due to oxidative reactions occurring during extended drying times. In contrast, microwave-vacuum drying better preserved phenolic compounds, as TPC showed no clear correlation with process temperature, reflecting the benefit of shortened drying times and reduced oxygen availability. Antioxidant capacity followed a similar trend, such that, under convective drying, the antioxidant values declined by nearly 32% when temperature increased from 50 to 90 °C, whereas microwave-vacuum dried samples maintained relatively stable antioxidant activity across power levels. Combined convective–microwave vacuum drying further reduced processing time while achieving phenolic and antioxidant retention comparable to microwave-vacuum drying alone, highlighting the advantage of hybrid approaches for stabilizing blackcurrant pomace with minimal bioactive degradation.

#### 3.4.5 Bignay pomace

Bignay (*Antidesma bunius*) berries are indigenous to the Philippines and the most dominant polyphenols in this berry are catechin (599.35 ± 2.79 mg/kg FM) and epicatechin (645.28 ± 4.10 mg/kg FM) (Carbonera et al., 2023). Comparison of convection oven-drying (45 °C, 48 h) with freeze-drying revealed marked differences in the preservation of polyphenols in bignay pomace (Table 3) (Zubia et al., 2023). Total phenolics were higher in freeze-dried pomace (1742 mg GAE/100 g DM) than in oven-dried samples (1273 mg GAE/100 g DM), and total anthocyanins followed a similar pattern (496 vs. 223 mg C3G/100 g DM). Flavonoids and tannins did not differ significantly between methods, suggesting that these more heat-stable classes withstand mild drying conditions. Antioxidant indicators were consistently higher in freeze-dried pomace, with ABTS +36%, DPPH +23%, and FRAP +7% relative to air-dried samples. Conversely, convection drying caused a 13% reduction in FRAP, reflecting partial degradation of redox-active phenolics. Catechin and epicatechin were identified as dominant flavonoids, and although the compound profile was unchanged, their concentrations were reduced after thermal treatment. These results confirm that low-temperature air drying, while practical, compromises total phenolic recovery relative to freeze-drying due to partial oxidation and polymerization of anthocyanin-rich compounds.

### 3.5 Cherry pomace

Sour cherry (*Prunus cerasus*) pomace, comprising skin and residual flesh and representing 15–28% of the original fruit mass, retains valuable constituents including polyphenols, dietary fiber, pectin, and minerals (Teslic et al., 2023). The phenolic composition and antioxidant properties of sour cherry pomace were evaluated by Ciccoritti et al. (2018), who compared freeze-drying and oven drying at 60 °C for 24 h in two cultivars: Bianchi d'Offagna and Montmorency. Total phenolic content was substantially higher in freeze-dried samples, reaching 45 ± 1 mg GAE/g DM for Bianchi d'Offagna and 19.0 ± 0.5 mg GAE/g DM for Montmorency, while oven drying reduced these values by 37–45%, confirming the adverse impact of prolonged thermal exposure on phenolic stability. Anthocyanins, dominated by cyanidin derivatives, were particularly sensitive to heat. Freeze-dried Bianchi d'Offagna pomace retained approximately 83% of TAC (485 mg C3G/100 g DM) compared with fresh pomace, whereas oven-dried samples lost over 65% of pigments. In Montmorency pomace, anthocyanin concentrations were markedly lower overall (∼7–8 mg C3G/100 g DM) and exhibited similar degradation patterns. Flavan-3-ols followed the same trend, decreasing by about 50% after oven drying, with freeze-dried Bianchi d'Offagna retaining 23.1 ± 0.3 mg catechin equivalents (CTE)/g DM versus approximately 11 mg CTE/g DM in the oven-dried counterpart. Antioxidant activity, measured by DPPH and ABTS assays, correlated strongly with phenolic and anthocyanin levels, and was consistently higher in freeze-dried samples. Overall, the study demonstrated that freeze-drying most effectively preserves the phenolic and pigment composition of cherry pomace, while conventional oven drying induces significant degradation due to thermal and oxidative stress. The results also highlight pronounced genotypic variation, with Bianchi d'Offagna exhibiting higher phenolic and anthocyanin levels than Montmorency, underscoring the importance of cultivar selection in optimizing cherry pomace valorization.

### 3.6 Peach and apricot pomaces

Peach (*Prunus persica*) and apricot (*Prunus armeniaca*) pomaces are rich reservoirs of phenolic compounds derived primarily from the peel and residual pulp. Extraction-based profiling of peach pomace shows that TPC typically ranges from 16.53 to 45.13 mg GAE/100 g FM, with compounds such as protocatechuic acid (up to 1.30 mg/100 g FM), chlorogenic acid, *p*-coumaric acid, caffeic acid, and especially apigenin (up to 5.41 mg/100 g FM) being prominent constituents (Baltacioglu et al., 2024). Apricot by-products similarly contain abundant hydroxycinnamic acids, particularly chlorogenic and neochlorogenic acids, with reported TPC ranging from 470 to 1290 mg GAE/100 g DW, depending on cultivar and extraction method (Kasapoğlu et al., 2021). Apricot pomace also contains flavonoids such as quercetin glycosides and catechin derivatives, together with appreciable antioxidant activity linked to these compounds.

The bioactive composition of peach and apricot pomaces is strongly influenced by drying temperature and duration, reflecting the thermosensitivity of both phenolic and carotenoid constituents. For peach pomace, Lalas et al. (2019) examined convective drying across 40–70 °C and found that both total phenolic content and carotenoids declined progressively with increasing temperature and exposure time. At the optimized condition of 70 °C for 8.27 h, total carotenoids reached 84.6 ± 8.6 μg β-carotene equivalents/g DM, corresponding to a 63% reduction compared with fresh pomace. Further heating beyond 70 °C resulted in additional carotenoid degradation exceeding 70%, accompanied by a parallel decrease in DPPH antioxidant activity of approximately 73%, demonstrating that the antioxidant potential of peach pomace is closely tied to its carotenoid fraction.

Similarly, apricot pomace exhibited temperature-dependent degradation of phenolic and carotenoid compounds under hot-air drying between 40 and 70 °C (Kayran & Doymaz, 2017). Although the study focused primarily on compositional changes, higher temperatures clearly reduced phenolic and carotenoid concentrations, consistent with oxidative breakdown of β-carotene and polyphenolic structures. Literature comparisons within the study indicated that moderate drying conditions (≈60 °C) better maintain phenolic content and antioxidant activity than either prolonged or higher-temperature treatments.

Collectively, these findings show that temperatures around 60–70 °C can achieve satisfactory dehydration of peach and apricot pomaces while partially preserving phenolic and carotenoid integrity. However, prolonged exposure or higher temperatures (>70 °C) markedly intensify oxidative degradation, leading to diminished antioxidant capacity. Thus, controlled thermal regimes that limit drying time and temperature are essential for maximizing the retention of bioactive compounds in *Prunus* pomaces.

### 3.7 Pear pomace

The pomace obtained from pear (*Pyrus communis*) contains a diverse profile of bioactive compounds, including 537–660 mg/100 g DW of total phenolics, 520–636 mg/100 g DW of flavonoids, 10.8–22.9 mg/100 g DW of carotenoids, and substantial amounts of tocopherols, tocotrienols, amino acids, and fatty acids (Ferreira et al., 2024). The influence of freeze-drying and contact-drying (40 °C + microwave 50 W, 60 °C, and 80 °C) on the phenolic composition and antioxidant properties of pear pomace has been evaluated (Krajewska et al., 2024). In this study, the freeze-drying process was performed in a contact-heat system with plate temperatures set at 20, 40, and 60 °C under a chamber pressure of 100 Pa; these values refer to the heating plate setpoints rather than the actual product temperature, which would remain below 0 °C during sublimation. TPC varied from 2.58 to 4.32 mg GAE/g DM, depending on the drying method and temperature. Freeze-dried pomace consistently retained higher phenolics (≈4.3 mg GAE/g DM) and showed stronger antioxidant activity (IC_50_ ≈ 44–45 mg DM/mL for both DPPH and ABTS) compared with contact-dried samples, which exhibited weaker radical scavenging efficiency (IC_50_ ≈ 71–92 mg DM/mL). The phenolic profile was dominated by quinic, chlorogenic, and protocatechuic acids. Contact-drying at 60 °C increased quinic and protocatechuic acid levels (198.9 and 4.2 mg/g DM, respectively), whereas freeze-drying at the same temperature favored chlorogenic acid (6.36 mg/g DM) and catechin derivatives (epicatechin ≈21.6 mg/g DM; catechin ≈0.43 mg/g DM). Gallic acid and epicatechin were substantially higher in freeze-dried samples, while they were undetected or drastically reduced under contact-drying, indicating thermal degradation of these more labile phenolics. Among flavonoids, isoquercitrin (4.0–13.9 mg/g DM) and rutin (0.9–2.7 mg/g DM) were the principal constituents, both enhanced under freeze-drying, particularly at 60 °C*. minor* flavonoids such as hesperidin, vanillin, and astragalin were detected in lower concentrations (<1 mg/g DM). These patterns confirm that freeze-drying better preserves quercetin glycosides and catechin derivatives, while contact-drying promotes partial oxidation and rearrangement of phenolic acids. In summary, freeze-drying at 20–40 °C produced pear pomace with the highest total phenolics and antioxidant activity, while contact-drying at 60–80 °C increased specific acids (quinic, protocatechuic) but at the cost of overall phenolic integrity. Thus, lyophilization remains the preferred approach for preserving both the quantity and diversity of phenolics in pear pomace, particularly when the goal is to maintain strong antioxidant potential and flavonoid bioactivity.

### 3.8 Orange and lemon pomaces

Mandarin orange (*Citrus reticulata*) pomace, consisting largely of peel, albedo and residual pulp, is characterized by high levels of dietary fiber, particularly pectin, as well as phenolics, flavonoids, and ascorbic acid. Afrin et al. (2022) reported total phenolic content of 10.4–19.3 mg GAE/g DM and total flavonoids up to ∼6 mg CE/g DM, with hesperidin and narirutin dominating the flavanone profile. In contrast, lemon pomace shows a similarly rich but distinct biochemical composition. According to Papoutsis et al. (2016), lemon (*Citrus limon*) by-products contain abundant pectin, high concentrations of flavanones such as eriocitrin and hesperidin, and a diverse range of phenolic acids, contributing to notable antioxidant capacity.

The drying behavior of citrus pomaces reflects distinct compositional and structural responses to temperature and oxygen exposure, with both orange and lemon species showing temperature-dependent degradation and transformation of phenolic compounds. For Mandarin orange, convective drying at 50–70 °C significantly influenced the retention of bioactive compounds (Afrin et al., 2022). TPC and TFC declined with increasing temperature, consistent with thermal oxidation of polyphenols and flavanones. Ascorbic acid content decreased from 119.36 to 110.23 mg/100 g DM between 50 and 70 °C, while antioxidant activity, measured as DPPH radical-scavenging capacity, dropped from 64.2% to 59.9% across the same range. These results highlight the progressive loss of both enzymatically sensitive and thermolabile compounds under extended exposure to warm air. Although phenolic degradation was moderate up to 60 °C, further heating to 70 °C intensified oxidative losses, suggesting that temperature control is critical for maintaining the antioxidant profile of orange pomace.

In lemon pomace, Papoutsis et al. (2017) observed a more complex trend across hot air, and VD conducted at 70–110 °C and freeze drying. TPC ranged between 15.8 and 20.7 mg GAE/g DM, with the highest values in hot air-dried samples at 110 °C. This increase was attributed to the thermal release of bound phenolic acids and enzyme inactivation during short-term exposure to high temperatures. TFC was best retained under VD at 70–90 °C, reaching approximately 4.6 mg CE/g DM, reflecting the protective role of reduced oxygen conditions. Among individual compounds, neohesperidin was most abundant in freeze-dried pomace (64.2 μg/mL), whereas rutin and *p*-coumaric acid peaked in vacuum-dried samples at 70 °C (137.0 μg/mL and 1.69 μg/mL, respectively). Conversely, gallic acid increased under hot air drying at 110 °C (8.7 μg/mL), possibly due to thermal cleavage of esterified forms. Antioxidant capacity, evaluated by DPPH and cupric ion reducing antioxidant capacity (CUPRAC) assays, showed the highest values for hot air and VD at 90–110 °C, surpassing freeze-drying. This enhancement likely reflects the greater extractability of heat-liberated phenolics rather than de novo formation of antioxidants. However, excessive heating or prolonged drying beyond 110 °C could accelerate flavonoid breakdown and darkening reactions.

In summary, both orange and lemon pomaces exhibit temperature-sensitive phenolic profiles. For orange pomace, temperatures ≤60 °C are most suitable to minimize losses of TPC, TFC, and vitamin C. In lemon pomace, moderate to high temperatures (70–90 °C) under vacuum or short-duration hot air drying enhance phenolic recovery by liberating bound acids and flavonoid glycosides. Collectively, these findings suggest that carefully balanced thermal regimes, combining moderate heat and limited oxygen exposure, are key to maximizing phenolic retention and antioxidant activity in citrus pomaces.

### 3.9 Kiwi pomace

Chinese kiwifruit (*Actinidia chinensis*) is notably rich in vitamin C, polyphenols, dietary fiber, minerals, and diverse phytochemicals such as triterpenoids and flavonoids, reflecting its high nutritional and bioactive potential (He et al., 2019). A study by Zhang et al. (2024) examined the influence of six drying methods: SD, HAD (60 °C), VD (60 °C, 0.087 MPa), MD, vacuum microwave drying (VMD, 60 °C, 0.087 MPa, 490 W), and VFD (−60 °C, 60 Pa) on the phenolic composition, antioxidant capacity, and fiber properties of Chinese kiwifruit pomace. The VFD samples exhibited the highest retention of phenolics and flavonoids, maintaining 85.8% and 90.7% of their original values, respectively. VMD achieved comparably high retention, while conventional HAD and SD showed the largest reductions, with TPC retention as low as 36.8–37.9% (Zhang et al., 2024). The improved preservation under VFD and VMD was attributed to reduced oxidation from oxygen exclusion and lower operating temperatures, whereas the longer exposure to air and heat in HAD and SD promoted polyphenol oxidase–mediated degradation. Antioxidant capacity followed the same trend as TPC: VFD > VMD > VD > MD > HAD ≈ SD. The high antioxidant potential in VFD and VMD samples reflected the strong retention of phenolics and flavonoids, while thermal processes such as HAD and SD caused substantial reductions due to prolonged oxidative and Maillard reactions.

Although total dietary fiber remained relatively stable across methods (54–56% of DM), drying significantly influenced the ratio of soluble to insoluble dietary fiber. Microwave-based drying (MD and VMD) yielded the highest soluble dietary fiber content (up to 9.5% DM), suggesting partial depolymerization of cellulose and hemicellulose into soluble fractions, while SD and HAD showed the lowest soluble fiber and hydration capacities. In contrast, VFD preserved cell wall integrity.

Collectively, VFD provided the best preservation of TPC, TFC, and antioxidant capacity. VMD, however, offered nearly equivalent quality with drastically reduced drying time and energy consumption, suggesting its suitability for industrial processing of kiwifruit pomace. In contrast, hot air and sun drying caused major polyphenol and pigment losses, limiting their use where functional quality is a priority.

## 4 Future perspectives, recommendations and overall insights

**Fig. 2:**
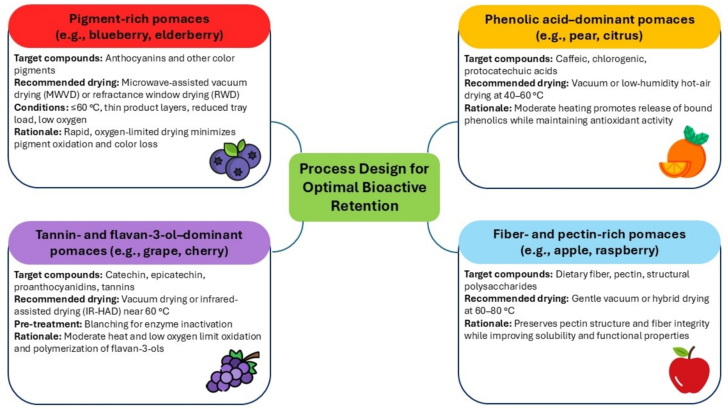
Process design recommendations for optimizing drying of fruit pomaces based on their dominant bioactive groups.

Considering all results together across fruits and drying technologies, several overarching trends emerge that can guide future research and industrial application. Across all matrices, the compositional profile of the pomace, whether dominated by anthocyanins, flavan-3-ols, or phenolic acids, determines its thermal and oxidative sensitivity, and consequently, the optimal drying approach; as summarized in Fig. 2.

Anthocyanin-rich pomaces such as blueberry, elderberry, bignay, and sour cherry consistently benefited from low-oxygen, short-residence drying techniques. In contrast, tannin- and flavan-3-ol–dominant systems, including grape and apple, tolerated moderate temperatures but were highly sensitive to oxidative conditions and prolonged residence times. Phenolic-acid–rich matrices such as citrus and pear occasionally exhibited higher extractable phenolics after drying due to the thermal release of bound forms; however, such increases were only advantageous under oxygen-restricted environments. These outcomes collectively highlight the need for matrix-aware process design rather than one-size-fits-all thermal guidelines.

Oxygen exposure and residence time repeatedly emerged as more decisive factors for bioactive preservation than temperature alone. In hot-air drying, reducing bed thickness and tray load, while increasing airflow uniformity, substantially improved the retention of phenolics and anthocyanins by promoting efficient moisture removal and limiting oxidative damage. In vacuum-based processes, such as vacuum-belt, microwave-assisted vacuum, and vacuum microwave drying, the combined effects of low oxygen partial pressure and rapid moisture transfer consistently resulted in superior preservation of phenolics, anthocyanins, and flavonoids. Notably, microwave-assisted processes only conferred advantages when coupled with vacuum conditions; microwave heating under atmospheric pressure, in contrast, often intensified oxidative degradation. Consequently, optimizing the triad of oxygen level, temperature, and time is more critical than focusing on any single parameter in isolation.

Hybrid drying represents one of the most promising strategies for reconciling product quality with process efficiency. Sequential systems that combine mild pre-drying with rapid oxygen-limited finishing, such as hot-air pre-drying followed by microwave-assisted vacuum or infrared drying, repeatedly achieved 50–90% reductions in total drying time while maintaining phenolic and anthocyanin levels close to those of freeze-dried samples. Refractance window drying, which offers uniform thin-film heating without requiring vacuum infrastructure, also proved highly effective for preserving pigments and phenolics, indicating its strong industrial potential where energy efficiency and equipment simplicity are priorities. These findings support the broader principle that rapid moisture removal in the final stages of drying is the most effective safeguard against degradation of thermolabile compounds.

Overall, a clear comparative pattern emerges across the available literature where the suitability of each drying technology is determined not only by its mode of heat and mass transfer, but also by the compositional profile of the pomace and the dominant bioactive fraction targeted for preservation. Conventional hot-air drying remains the most accessible and scalable option, particularly where moderate phenolic retention is acceptable and cost-effective stabilization is the primary objective, but it is limited by long residence times and high oxidative exposure. Freeze-drying provides the highest preservation of thermolabile compounds and structural integrity, yet its high energy demand and limited industrial throughput restrict broader application. Vacuum-based systems offer a more favorable balance between bioactive retention and process feasibility by reducing oxygen exposure and lowering thermal load, while microwave-assisted systems are especially effective where rapid moisture removal is required, provided that heating uniformity is maintained. Infrared and refractance window drying are most advantageous for thin-layer materials and pigment-rich matrices because of their rapid heat transfer and reduced exposure time. Hybrid systems appear most versatile overall, as they combine complementary advantages of individual methods; however, their effectiveness remains strongly matrix-dependent and requires careful optimization of time–temperature–oxygen interactions.

Beyond phenolics, several studies underline the importance of simultaneously considering fiber and pectin quality, especially in matrices such as apple, kiwi, and raspberry where polysaccharide valorisation is relevant. In apple pomace, pectin's galacturonic acid content was stable across moderate drying temperatures, but excessive heat reduced its molecular weight and degree of methoxylation, impairing gelling performance. Vacuum drying preserved total dietary fiber at levels comparable to freeze-drying, maintaining cell-wall integrity and enabling downstream pectin extraction. Similarly, moderate thermal and microwave-assisted drying increased soluble dietary fiber in raspberry and kiwi pomaces without compromising phenolic content, suggesting opportunities to co-optimize antioxidant and fiber properties in future process designs.

A major limitation in current research lies in the inconsistency of reporting and experimental standardization. This heterogeneity also limits direct quantitative comparison across studies, necessitating cautious interpretation of absolute values. To enable meaningful comparisons and data synthesis, drying studies should systematically specify whether results are expressed on a dry or fresh mass basis, define the analytical equivalents used for phenolic quantification, and detail extraction and assay conditions. Similarly, comprehensive process data, including temperature profiles, pressure levels, air velocity, tray load, microwave power density, relative humidity, residence time, endpoint moisture, and water activity, should be routinely reported. Incorporating full phenolic profiles, rather than only total sums, would allow for better understanding of class-specific degradation pathways and facilitate modeling of drying kinetics. The establishment of harmonized analytical protocols and reporting standards is therefore essential to enable robust cross-study comparison and data integration.

Future research should prioritize kinetic modeling that explicitly integrates oxygen concentration, residence time, and temperature to predict degradation of key bioactive compounds. In microwave-based systems, the relationship between power density, internal temperature distribution, and bioactive retention warrants further examination to support scale-up and industrial optimization. Systematic evaluation of hybrid drying sequences, holding final water activity constant, would help distinguish thermal from time-dependent effects. Furthermore, multi-objective optimization approaches that consider both antioxidant compounds and polysaccharide quality (particularly soluble dietary fiber and pectin molecular characteristics) would provide a more holistic view of process performance for fruit pomaces like apple pomace. Finally, future work should include energy-use data to assess the environmental and economic trade-offs of emerging technologies relative to conventional methods. In addition, advanced predictive tools, including machine learning and AI-assisted optimization, may support the development of matrix-specific drying protocols by integrating multiple process variables and quality targets. Such approaches could be particularly valuable for identifying optimal drying strategies that balance bioactive retention, energy efficiency, and industrial feasibility.

From a practical perspective, process design should always be guided by the dominant bioactive group and intended end use. For anthocyanin-rich pomaces, such as blueberry, elderberry, bignay, and sour cherry, the optimal strategy is low-oxygen and short-residence drying, with microwave-assisted vacuum, vacuum drying, refractance window drying, or carefully optimized hybrid systems preferred over prolonged hot-air drying. When convective drying is applied to such matrices, temperatures near 60–70 °C, combined with reduced tray load and thin product layers, are more suitable for limiting pigment degradation. For tannin- and flavan-3-ol–rich pomaces, particularly grape and apple, moderate-temperature vacuum drying or infrared-assisted drying near 60 °C appears most appropriate, while enzymatic inactivation steps such as blanching may further reduce oxidation where relevant. For phenolic-acid–rich matrices, including some citrus and pear pomaces, moderate heating under oxygen-limited conditions may be advantageous because it can enhance the release of bound phenolics without severely compromising antioxidant activity. For pomaces in which preservation of fiber and pectin functionality is a major objective, such as apple, raspberry, and kiwi, gentle vacuum or hybrid drying in the range of approximately 60–80 °C provides the most balanced approach by achieving sufficient moisture removal while minimizing structural damage to the polysaccharide matrix. In conclusion, the optimal drying strategy is not a single technology, but a matrix-specific balance between oxygen control, residence time, drying rate, and targeted quality attributes.

## 5 Conclusions

Across the diverse range of fruit pomaces evaluated, the preservation of phenolic and flavonoid compounds depends less on the nominal drying temperature than on the interplay of oxygen exposure, heating rate, and total residence time. Conventional hot-air drying can achieve satisfactory retention of antioxidants when conducted at 60–70 °C with thin layers, short processing times, and adequate airflow. However, low-oxygen and rapid-transfer methods (vacuum, microwave-assisted vacuum, infrared, and refractance window drying) more consistently preserve total phenolic, flavonoid, anthocyanin, and tannin contents while maintaining the structural integrity of fiber and pectin where applicable. Apple exemplifies the dual objective of preserving both antioxidants and the polysaccharide framework for integrated valorisation; grape highlights the susceptibility of tannins and flavan-3-ols to oxidative polymerization; blueberry, elderberry, bignay, and cherry demonstrate the fragility of anthocyanins; cranberry underscores the influence of tray load and airflow on pigment stability; and kiwi and raspberry illustrate how controlled microwave or vacuum conditions can enhance soluble fiber without compromising bioactive retention. Overall, future research should move beyond isolated case studies toward matrix-specific, standardized, and scalable drying frameworks. Designing processes that minimize oxygen exposure and total drying time, while explicitly optimizing for both bioactive and structural quality, represents the most promising route to producing high-value, functional ingredients from fruit-processing by-products within a circular bioeconomy framework.

## Declaration of generative AI and AI-assisted technologies in the manuscript preparation process

During the preparation of this work the authors used OpenAI's ChatGPT 5.0 in order to enhance the readability and language of the text. After using this tool, the authors reviewed and edited the content as needed and take full responsibility for the content of the published article.

## CRediT authorship contribution statement

**Ali Ali Redha:** Writing – review & editing, Writing – original draft, Visualization, Investigation, Conceptualization. **Aysun Yücetepe:** Writing – review & editing, Writing – original draft. **Tuba Esatbeyoglu:** Writing – review & editing, Supervision, Funding acquisition, Conceptualization.

## Declaration of competing interest

The authors declare the following financial interests/personal relationships which may be considered as potential competing interests: Given her role as Editor, Tuba Esatbeyoglu, had no involvement in the peer-review of this article and has no access to information regarding its peer-review.

## Data Availability

The data presented in this study are available in the referenced articles.
